# Contaminants in fish from U.S. rivers: Probability-based national assessments

**DOI:** 10.1016/j.scitotenv.2022.160557

**Published:** 2022-11-28

**Authors:** Leanne L. Stahl, Blaine D. Snyder, Harry B. McCarty, Thomas M. Kincaid, Anthony R. Olsen, Tara R. Cohen, John C. Healey

**Affiliations:** a U.S. Environmental Protection Agency, Office of Water/Office of Science and Technology, 1200 Pennsylvania Avenue, NW (MC 4305T), Washington, DC 20460, USA; b Tetra Tech, Inc., Center for Ecological Sciences, 10711 Red Brook Boulevard, Suite 105, Owings Mills, MD 21117, USA; c General Dynamics Information Technology, 3170 Fairview Park Drive, Falls Church, VA 22042, USA; d U.S. Environmental Protection Agency, Office of Research and Development, Center for Public Health and Environmental Assessment, Pacific Ecological Systems Division, 200 S.W. 35^th^ Street, Corvallis, OR 97333, USA; 1 Retired.

**Keywords:** Fish tissue, Mercury, Polychlorinated biphenyls, PCBs, Per- and polyfluoroalkyl substances, PFAS

## Abstract

Most fish consumption advisories in the United States (U.S.) are issued for mercury and polychlorinated biphenyls (PCBs), and recently per- and polyfluoroalkyl substances (PFAS) have become a contaminant group that warrants fish consumption advice. An unequal probability survey design was developed to allow a comprehensive characterization of mercury, PCB, and PFAS contamination in fish from U.S. rivers on a national scale. During 2013–14 and 2018–19, fish fillet samples were collected from 353 and 290 river sites, respectively, selected randomly from the target population of rivers (≥5th order in size) in the conterminous U.S. These comprised nationally representative samples, with results extrapolated to chemical-specific sampled populations of 48,826–79,448 river kilometers (km) in 2013–14 and 66,142 river km in 2018–19. National distribution estimates were developed for total mercury, all 209 PCB congeners, and up to 33 PFAS (including perfluorooctane sulfonate or PFOS) in river fish. All fillet tissue samples contained detectable levels of mercury and PCBs. One or more PFAS were detected in 99.7 % and 95.2 % of the fillet samples from fish collected in 2013–14 and 2018–19, respectively. Fish tissue screening levels applied to national contaminant probability distributions allowed an estimation of the percentage of the sampled population of river lengths that contained fish with fillet concentrations above a level protective of human health. Fish tissue screening level exceedances for an average level of fish consumption ranged from 23.5 % to 26.0 % for mercury, 17.3 % to 51.6 % for PCBs, and 0.7 % to 9.1 % for PFOS.

## Graphical Abstract

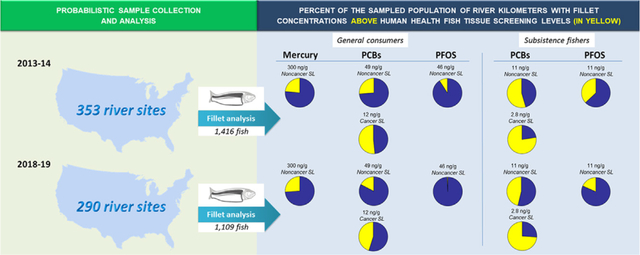

## 1. Introduction

State health protection and natural resource agencies in the United States (U.S.) issue fish consumption advisories to protect people from potential risks associated with eating contaminated fish from local waters. In 2011, 36 % (or 2.19 million kilometers [km]) of the total U.S. river km were under active fish consumption advisories for >30 contaminants (USEPA, 2013a), but mercury and polychlorinated biphenyls (PCBs) accounted for 89 % of all advisories. Based on national probabilistic survey results (USEPA, 2009; Stahl et al., 2009), nearly half (48.8 %) of the sampled population of lakes in the conterminous U.S. had fish with mercury concentrations that exceeded the U.S. Environmental Protection Agency’s (EPA’s) 300 nanograms (ng) per gram (g) fish tissue-based water quality criterion (USEPA, 2001b). Walters et al. (2010) and Riva-Murray et al. (2020) noted that mercury fish tissue studies have often focused on lentic waters; therefore, more information is needed on the spatial patterns and temporal changes in mercury concentrations in fish from flowing water (lotic) habitats.

Mercury is widely recognized as a persistent, bioaccumulative, and toxic (PBT) contaminant in aquatic ecosystems. Microbial transformation of elemental mercury into methylmercury in the aquatic environment increases its toxicity and bioavailability, resulting in biomagnification from primary producers (e.g., algae), to fish, to top piscivores including humans (Sweet and Zelikoff, 2001). Aquatic food web research has shown that this organic form of mercury can biomagnify in predator fish to levels that are seven orders of magnitude higher than concentrations in the source water (Wiener et al., 2003); therefore, fish consumption can be a primary pathway of mercury exposure for many human populations (Driscoll et al., 2013). The human health risks associated with elevated mercury exposure include impaired neurodevelopment (Mergler et al., 2007; Rice, 2008) and an increased risk of cardiovascular disease (Guallar et al., 2002). The health benefits of eating fish are widely recognized and advocated; however, fish consumption recommendations or limitations are still warranted to protect humans from exposure to PBT chemicals and their associated health risks.

Although the production of PCBs was banned in the U.S. in 1979, studies show that they are still widely distributed and extremely persistent in the environment (USEPA, 2009; Blocksom et al., 2010; Chang et al., 2012). Due to their hydrophobic nature, most PCBs in aqueous environments will bind to particle matter that settles into the sediment layer; however, some PCBs can remain dissolved in water. PCBs are lipophilic compounds, they biomagnify through the food web, and as a result, their levels in aquatic biota can be as much as six orders of magnitude higher than levels in the aquatic environment (ATSDR, 2000). Maternal consumption of PCB-contaminated fish can cause reproductive complications and neurobehavioral impacts in newborns and children (ATSDR, 2014), and neurological effects can be directly related to the frequency of fish consumption (Mergler et al., 1998). The potential adverse human health effects of low-level environmental PCB exposure are complex, requiring further evaluation; therefore, continued monitoring of PCBs in the environment is warranted.

In addition to mercury and PCBs, an increasing number of fish contamination studies and state surveillance programs are now assessing other bioaccumulative compounds of concern, including per- and polyfluoroalkyl substances (PFAS), with a specific focus on perfluorooctane sulfonate (PFOS) (Stahl et al., 2014). PFAS comprise a group of many organofluorine chemicals that are of concern because of their toxicity, widespread distribution, and persistence in the environment (Lindstrom et al., 2011). They bioaccumulate through food webs, have a long half-life in humans, bind to blood and liver proteins in fish and mammals, and do not demonstrate lipophilic properties that are characteristic of many other organic contaminants (Jones et al., 2003; Conder et al., 2008; Houde et al., 2011). Researchers in New York State found average PFOS concentrations in fish to be 8850-fold greater than levels in surface waters (Sinclair et al., 2006). Human epidemiology studies have reported associations between PFAS exposure and developmental effects (e.g., decreased birth weight), decreased immunity, and cardiovascular effects, as well as evidence suggesting carcinogenic potential in humans (USEPA, 2022a). Studies suggest that the consumption of fish from contaminated waters may be a major source of PFOS and long-chain PFAS exposure for some people (Christensen et al., 2017; Augustsson et al., 2021). Alabama, Michigan, Minnesota, and Wisconsin all had active PFOS fish advisories in 2016 and released PFOS-based fish consumption advice to the public within their jurisdictions (USEPA, 2016a). New York and New Jersey issued their first PFOS-related fish consumption advisories in 2017 and 2018, respectively. In 2021, Maryland issued their first PFOS-based fish consumption advisory, and Massachusetts set fish consumption limits for five lakes on Cape Cod based on PFAS monitoring results.

Many biological monitoring and health assessment studies have investigated PBT contaminants of concern in U.S. fish; however, most were limited in scale (e.g., local site or state political boundaries) and/or focused on areas of known (or perceived) contamination. To broaden contemporary knowledge regarding contamination in fish from U.S. rivers, EPA conducted a comprehensive characterization of mercury, PCB, and PFAS fish fillet tissue contamination on a national scale using an unequal probability design developed for the agency’s National Rivers and Streams Assessment (NRSA) (USEPA, 2015a). The NRSA framework included a statistically representative subset of river sites that were 5th order or greater in size based on Strahler stream order classification (Strahler, 1957) and had fish present that are commonly consumed by humans. For size perspective, Downing et al. (2012) noted that 5th order rivers have an average width and length of 29 m and 45 km, respectively. In the 2013–14 study, a total of 353, 223, and 349 fish composite samples were analyzed for mercury, PCBs, and PFAS, respectively; a total of 290 samples were analyzed for all target chemicals in the 2018–19 study. Results from multiple study years allow the first national temporal comparison of fish contaminant levels in rivers. Mercury results for 541 fish fillet samples were also available from the 2008–09 NRSA (Wathen et al., 2015; USEPA, 2016b), allowing a 10-year temporal comparison to 2018–19 results. The objective of the NRSA fillet fish tissue studies was to estimate the national distribution of the mean levels (i.e., composite average) of mercury, PCBs, and PFAS in the fillet tissue of fish from rivers of the conterminous U.S., to compare them to fish tissue screening levels (Table A.1) for human health impact assessment, and to highlight temporal changes in fish contaminants in U.S. rivers.

## 2. Methods

### 2.1. Probability design and site selection

To be included in the NRSA fish fillet tissue study target population, rivers had to be located in the conterminous U.S., be ≥5th order in size, have a permanent fish population, and have flowing water during the sampling period. Great rivers and run-of-the-river ponds were included. Reservoirs and portions of tidal rivers up to head-of-salt were excluded. Strahler stream order attributes found in the National Hydrography Dataset (NHD) and land use attributes from NHD-Plus (USGS and USEPA, 2010) and from the U.S. Census Bureau national urban boundary geographic information system (GIS) coverages were used to derive the sample frame. The process for delineating urban versus nonurban areas (polygons) is described in Stahl et al. (2014).

Sites selected for these fish tissue studies are a subset of the sites selected for the NRSA to study a broad array of ecological indicators in rivers and streams in the conterminous U.S. (https://www.epa.gov/national-aquatic-resource-surveys/nrsa). The NRSA probability-based survey design is stratified by state and by nine aggregated ecoregions (Omernik, 1987); within each stratum, sites were selected using unequal probability selection classes of small streams (1st, 2nd order), large streams (3rd, 4th order), and rivers (≥5th order). The allocation of sites to states was proportional based on stream and river length within the strata. Site selection for the fish contamination studies was restricted to the part of the NRSA target population that included rivers that were 5th order or greater and met the other requirements described above. Approximately 50 % of the sampling sites were selected from those sampled during a previous NRSA study, and 50 % were new sites for each study. Both sets of sites followed the same stratification and unequal probability selection classes. Within each stratum, the sites were selected using a spatially balanced approach, termed a Generalized Random Tessellation Stratified (GRTS) survey design by Stevens and Olsen (2004). Use of the probability-based survey design assured that every location on rivers had an opportunity to be selected with a known probability, which consequently allowed inferences to the entire sampled population of rivers that are included in these fish contamination studies.

**Fig. 1.**
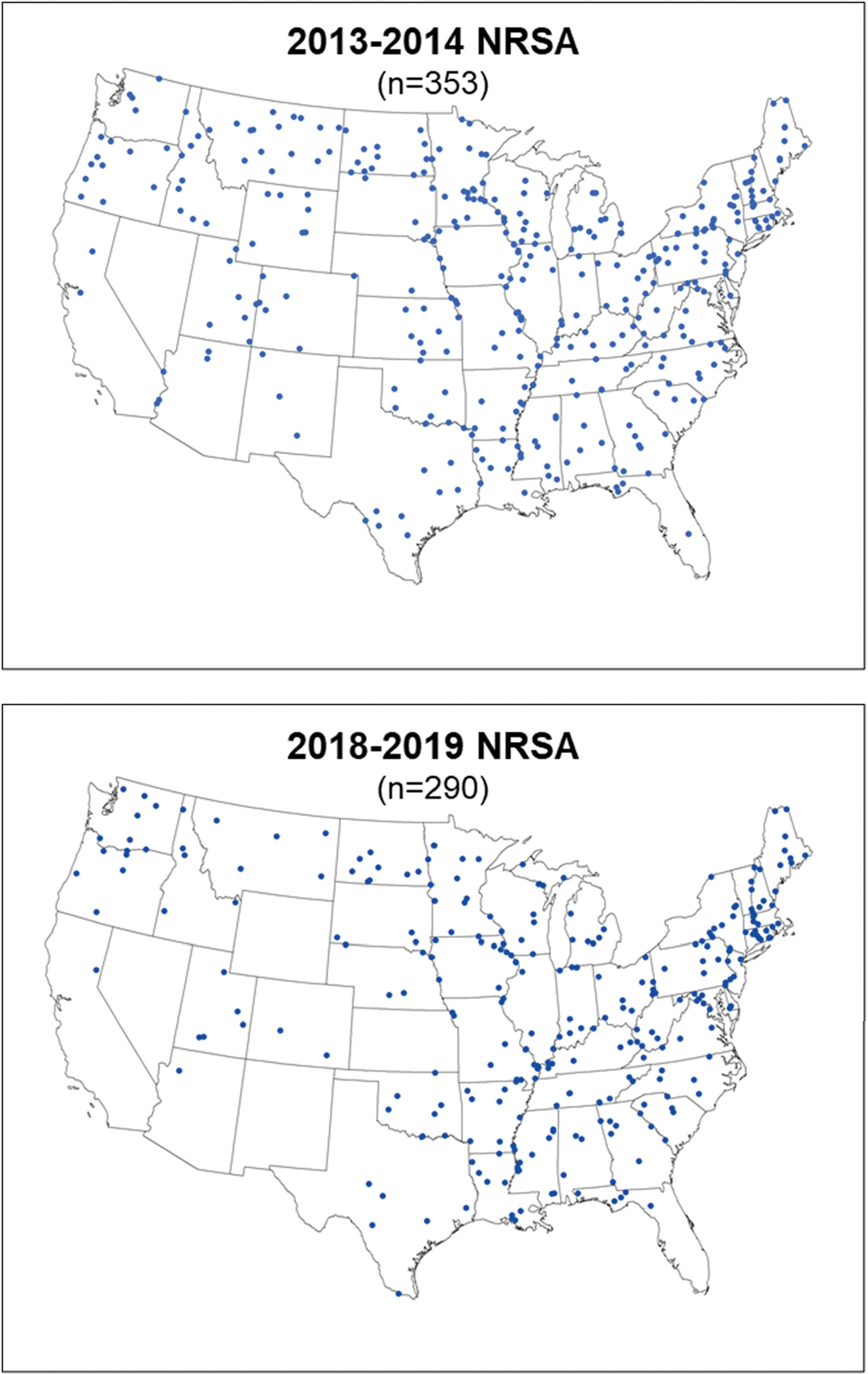
National Rivers and Streams Assessment 2013–14 and 2018–19 fish sampling locations.

A probability-based design was essential because the primary purpose was to describe fish contaminant levels on a nationally representative basis. A key element of the survey design was the inclusion of the strata associated with previous NRSA studies. This component included a subset of sampling locations that was selected and successfully sampled in the 2008–09 NRSA, then also sampled during subsequent studies to allow for estimation of change in fish contaminant levels between points in time (in this case over 5 or 10 years). Fish samples were collected from randomly selected river sites in 48 and 45 states during the 2013–14 and 2018–19 study periods, respectively (Fig. 1).

### 2.2. Sample collection

Fish targeted for collection were specified in EPA field procedures and were known to be primarily predator species (in many cases top carnivores) that are ubiquitous, abundant, commonly consumed by humans, and sufficiently large to provide adequate tissue for analysis (USEPA, 2013b, 2019a). One fish composite sample was collected from each site primarily by electrofishing during survey periods in April through October in 2013–14 and May through November in 2018–19. Field crews were directed to attempt to collect a composite sample containing five adult fish, and they were alerted that it was critical that all fish in the composite be the same species and be comparable in size (i.e., the smallest specimen in a composite must be at least 75 % of the total length of the largest individual, following USEPA, 2000a). It was less critical that composite samples contain five fish, so composites with fewer than five fish were accepted for analysis to obtain fillet tissue data from each target river site. Similarly, composite samples comprising more than five small fish were accepted to have sufficient fillet tissue for contaminant analyses. Field crews packed the whole fish from each site in a cooler containing dry ice and shipped coolers to the laboratory under contract to EPA for sample storage and preparation (i.e., filleting and homogenization) prior to analysis. Fish collection and field sample handling procedures are specified in field operation manuals (USEPA, 2013b, 2018a), and laboratory sample handling and preparation requirements are detailed in quality assurance project plans (USEPA, 2015b, 2018b).

### 2.3. Sample preparation and analysis

Fillet tissue sample preparation procedures followed a standardized approach (USEPA, 2015b, 2018b). Quality control (QC) steps were integrated into these procedures, including testing the homogeneity of the fillet tissue samples and testing the equipment used in the preparation of each sample batch for contamination. To process each composite sample, laboratory staff scaled the fish, removed skin-on fillets from both sides of each fish with the ventral muscle (belly flap) attached, and used a grinder to homogenize all the fillet tissue from each fish in the composite (an approach referred to as the batch method in USEPA, 2000a). Aliquots were removed for mercury, PCB, and PFAS analysis and subsequently shipped to analytical laboratories. In each of the NRSA studies, only one laboratory was contracted for the analysis of a given class of contaminants, thereby eliminating between-laboratory variability within a given study.

Fish fillet samples were prepared for total mercury analysis using Procedure I from “Appendix to Method 1631, Total Mercury in Tissue, Sludge, Sediment, and Soil by Acid Digestion and BrCl Oxidation” from Method 1631 Revision B (USEPA, 2001a) and were analyzed for mercury using Method 1631 Revision E (USEPA, 2002). This method required approximately 1 g of homogenized tissue for analysis, digestion with a combination of nitric and sulfuric acids, oxidation with bromine monochloride (BrCl), and analysis by cold-vapor atomic fluorescence spectrometry.

Separate laboratories analyzed the PCB aliquots in 2013–14 and 2018–19; however, both laboratories utilized EPA Method 1668 Revision C, “Chlorinated Biphenyl Congeners in Water, Soil, Sediment, Biosolids, and Tissue by HRGC/HRMS” (USEPA, 2010a). Samples were analyzed for all 209 PCB congeners and reported as either individual congeners or coeluting groups of congeners (Table A.2). The laboratories employed minor modifications to Method 1668C, which are detailed in the respective analytical quality assurance project plans (USEPA, 2015b, 2019b) and fall within the method’s established allowance for flexibility. All modifications were reviewed and accepted for the purposes of these studies.

There were no standardized analytical methods for PFAS analysis of a tissue matrix from EPA or any voluntary consensus standard bodies at the time of these studies; therefore, EPA relied on procedures developed by the two commercial laboratories that conducted PFAS analysis. The number of PFAS analytes tested in the two studies increased from 13 strictly perfluorinated compounds in 2013–14 to 33 PFAS in 2018–19, representing a larger number of functional groups attached to the carbon chains. This change was a function of both the larger number of native and labeled standards that became available from commercial suppliers by 2018 and the recognition that many PFAS were present in the environment. Because the fillet tissue samples were analyzed for PFAS using similar procedures that are currently considered proprietary by both laboratories, they can be described only briefly. In both studies, the laboratories prepared between 1 g and 5 g of homogenized fish fillet tissue for PFAS analysis. Samples were spiked with 12 isotopically labeled standards in 2013–14 and 24 labeled standards in 2018–19, then they were extracted by shaking the tissue in a caustic solution of methanol, water, and sodium or potassium hydroxide. After extraction, the laboratories centrifuged the solution to remove the solids, diluted the supernatant liquid with reagent water, and processed the diluted extract by solid-phase extraction (SPE). After the PFAS were eluted from the SPE cartridge, the eluant was spiked with additional labeled recovery standards and analyzed by high performance liquid chromatography with tandem mass spectrometry (HPLC-MS/MS) for the PFAS listed in Table A.3.

These analytical procedures yielded results for 13 and 33 PFAS in their anion forms (e.g., C_3_F_7_COO−) for the 2013–14 study and 2018–19 study, respectively; however, the results in Table A.3 are reported for the parent acid forms (e.g., C_3_F_7_COOH). The concentration of each PFAS is determined using the responses from one of the ^13^C- or ^18^O-labeled standards added prior to sample extraction by applying isotope dilution quantitation. That quantitation approach is fundamental to many of EPA’s 1600-Series analytical methods, including methods for PCBs, dioxins and furans, semivolatile organics, and other contaminants (for example, see USEPA, 2010a). As a result, all the target analyte concentrations are corrected for the recovery of the labeled standards, thus accounting for extraction efficiencies and losses during cleanup.

As described in Stahl et al. (2014), all the analytical procedures employed in these studies involved extensive QC operations, including method blanks, laboratory control samples, multi-point initial calibrations, and daily calibration verifications, and acceptance criteria were specified for each operation (USEPA, 2002, 2010b, 2015b, 2019b). All analytical results are expressed as wet-weight concentrations in ng/g and are reported down to the Method Detection Limit (MDL).

### 2.4. Data analysis

Data analysis focused on the study objective to develop estimates of the national distribution of mean levels (i.e., composite average concentrations) of PBT contaminants in river fish tissue.

#### 2.4.1. Probability distribution of target analytes

Total mercury, total PCB, and PFOS data were analyzed using R statistical software (R Core Team, 2015) and the package “spsurvey 4.1.4” (Kincaid and Olsen, 2020). The stratified, unequal probability survey design for site selection (Olsen et al., 2009) requires incorporation of the survey design weight associated with each site in the statistical analysis. The weight is the inverse of its inclusion probability for the survey (Lohr, 1999). The weight depends on whether the site was sampled in a previous NRSA study or is a new site, the aggregated ecoregion it was in, its stream order category, and the state it was in. For each combination, the weight is the river length within the combination divided by the number of sites evaluated within the combination. The process for analyzing fillet tissue results from a probabilistic sampling design involves five fundamental steps, including: (1) determining the status of each site using site evaluation information; (2) applying the site status information to revise the design weights to account for the design as implemented; (3) estimating length of rivers in the target population based on the number of sites that met the study definition of a river; (4) generating estimates of the river length and proportion of river length in the sampled population, which represents the total length associated with accessible river segments that were successfully sampled for fish; and (5) producing cumulative distribution functions of fillet tissue contaminant concentrations for the sampled population of river length. The resulting total mercury, total PCB, and PFOS distributions are described by study using estimates of percentiles and cumulative distribution functions (CDFs) (Zar, 1999; Kincaid and Olsen, 2020). The CDFs characterize the probability distribution of the target analytes in fish fillet tissue as plots of concentrations (x-axis) versus the cumulative number of river km and percentage of the sampled population (y-axis).

#### 2.4.2. Fish tissue screening levels

USEPA (2000b) describes basic equations used to develop the minimum contaminant concentrations for evaluating potential long-term human health impacts from fish consumption, which are referred to as fish tissue screening levels (Table A.1). The percentage of the sampled population of U.S. river km containing fish with fillet concentrations that exceed a level for human health protection can be estimated by overlaying these screening levels on CDF plots. The screening level used for interpreting human health risks related to fillet mercury concentrations was EPA’s fish tissue-based criterion for methylmercury (USEPA, 2001b). PCB risk-based screening levels were calculated for cancer and noncancer effects using equations provided in USEPA (2000b), along with a nutrition-based fish consumption rate of 32.4 g/day for general fish consumers (representing one 8-ounce meal per week), and an elevated fish consumption rate of 142 g/day for high-frequency fish consumers (i.e., those who eat four to five 8-ounce meals per week, hereafter referred to as subsistence fishers). Risk-based screening levels applied to PFOS concentrations in fish fillet tissue were derived using EPA noncancer health effects equations (USEPA, 2000b), the human health reference dose (RfD) for PFOS published in USEPA (2016c), current values for pregnant adult body weight (USEPA, 2011), and fish consumption rates for general fish consumers and for subsistence fishers. In 2022, EPA issued interim drinking water health advisories for perfluorooctanoic acid (PFOA) and PFOS based on an evaluation of over 400 human epidemiology studies published from 2016 to 2021 and new modeling approaches (USEPA, 2022b, 2022c). Those analyses are undergoing Science Advisory Board review. EPA’s interim noncancer RfD for PFOS is 7.9 × 10^−9^ mg per kilogram per day (mg/kg-day), four orders of magnitude lower than EPA’s 2016 RfD. A decrease in the PFOS RfD would also decrease the fish tissue screening level and would, therefore, increase the number of PFOS exceedances above the revised fish tissue screening level when compared to the number of exceedances reported for these studies.

#### 2.4.3. Analysis of temporal changes

The final step in the analysis of fish fillet data addressed the objective for both studies of tracking changes in contaminant levels in fish fillet tissue over time. This step involved estimating change in the percentage of the sampled population of U.S. river km containing fish with fillet contaminant concentrations that exceeded fish tissue screening levels during the 5-year interval between the two studies. These change estimates were derived using the “spsurvey 4.1.4” package in R (Kincaid and Olsen, 2020). Statistically significant changes in the percentage of the sampled population with fish tissue contaminant screening level exceedances occurred when the range for the error bars around the change estimates did not include zero.

## 3. Results and discussion

### 3.1. Sampling results

The 2013–14 and 2018–19 river sampling locations (Fig. 1) are national random samples and constitute statistically representative subsets of the rivers in the conterminous U.S. (i.e., ≥5th order). Fish collection in 2013–14 yielded 353 composite samples (1416 total fish) of 37 species, and sampling in 2018–19 yielded 290 composite samples (1109 total fish) of 37 species (Table A.4). Target fish species and size recommendations were based on guidance in USEPA (2000a), focused on species commonly consumed by humans, and were clearly identified in study-specific procedures (USEPA, 2013b, 2018a); however, the outcome of sampling efforts ultimately depended on the natural diversity and abundance of fish at each sampling location. Three of the recommended target species—Channel Catfish (*Ictalurus punctatus*), Smallmouth Bass (*Micropterus dolomieu*), and Largemouth Bass (*Micropterus salmoides*)—predominated in both surveys, accounting for 53 % of the samples in 2013–14 and 54 % of the samples in 2018–19.

### 3.2. Analytical quality control results

Samples were digested (mercury) or extracted (PCBs and PFAS) and analyzed in batches of up to 20, with each batch having its own method-specific QC samples. All mercury QC analyses in both studies met the acceptance criteria (Table A.5).

PCB QC analyses are summarized in Table A.6. Despite extensive efforts by laboratories, low levels of PCB congeners are common occurrences in method blanks, especially when using sensitive analytical methods. A total of 2 % of the 36,512 PCB congener results in the 2013–14 study and 6 % of the 46,400 results in the 2018–19 study were associated with method blanks in which one or more congeners were detected. When the analyte concentration in a sample is less than five times its concentration in the associated method blank, the risk of a false positive result or inflated sample concentration is considered great enough that the analyte was treated as not detected in the sample, affecting 0.6 % and 0.9 % of the results in 2013–14 and 2018–19, respectively.

PFAS are also common in the environment, including the laboratory, where analytical equipment may have parts containing polytetrafluoroethylene which must be removed to conduct PFAS analyses. In the 2013–14 and 2018–19 studies, 8 % and <1 % of the results, respectively, were associated with a method blank in which one or more analytes were detected (Tables A.7 and A.8). The decrease between studies likely reflects ongoing efforts to identify and minimize sources of PFAS in analytical laboratories in general. PFAS results that were less than five times the associated method blank result were treated as not detected in the sample, affecting 2.7 % of the 2013–14 results and 0.5 % of the 2018–19 results.

In addition to increasing the number of target PFAS to 33 in the 2018–19 study, the laboratory also added a large number of labeled compounds to quantify the new target analytes. Those additions, combined with a narrowing of the QC limits for labeled compound recovery, led to differing labeled compound recovery issues observed between the two studies (Tables A.7 and A.8).

### 3.3. Analytical results

The 2013–14 fillet composites analyzed for each chemical or group of chemicals comprised a nationally representative sample whose results can be applied to the sampled populations with an estimated river length of 79,448 km for mercury; 48,826 km for PCBs; and 78,272 km for PFAS. The differences in the sampled populations reflect the different number of samples analyzed for each chemical or group of chemicals. The composite samples collected during 2018–19 were analyzed for all target chemicals and therefore results can be applied to a sampled population of 66,142 river km. All the fish fillet samples from both studies contained detectable levels of mercury and PCBs. PFAS were detected in 99.7 % and 95.2 % of the total samples analyzed in 2013–14 and 2018–19, respectively. Analytical results and statistical estimates for mercury, PCBs, and PFAS in fillet samples are presented in Tables 1, 2, and 3, respectively, as minimums, maximums, and percentiles (ng/g, wet weight), and are reported to three significant figures.

#### 3.3.1. Mercury detections and concentrations

**Fig. 2.**
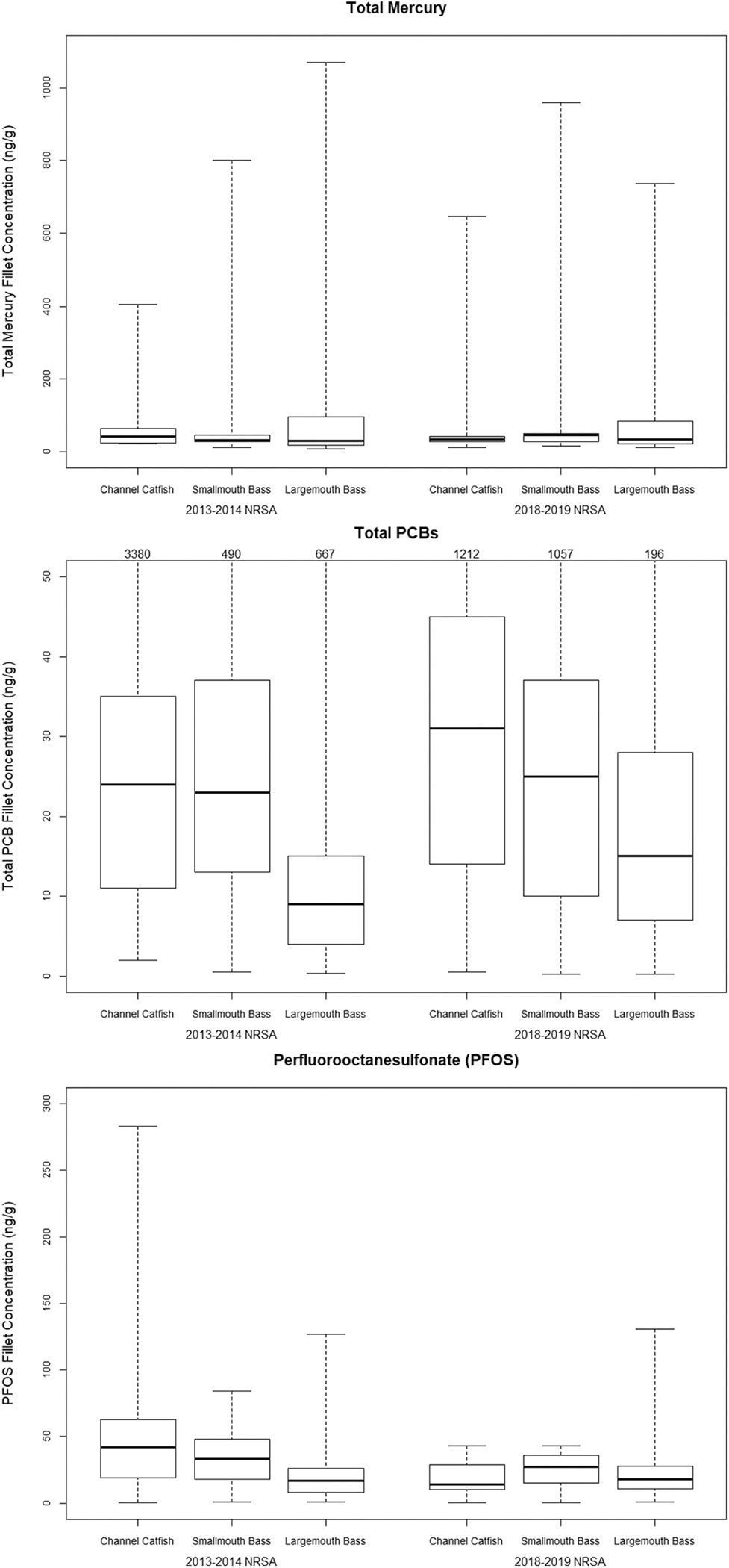
Minimum, maximum, median, and interquartile range (25th and 75th percentiles) of total mercury, total PCB, and PFOS (ng/g, wet weight) concentrations detected in fillet tissue of the predominant river fish species collected for the 2013–14 and 2018–19 NRSA fish tissue studies (a close-up view is provided for PCBs to increase the visibility of the interquartile ranges, and maximum values are listed at the top of each plot).

Freshwater fish contamination studies have shown that methylmercury can account for >90 % of the total mercury concentration in predator fish tissue (Bloom, 1992; USEPA, 2010b). In 2000, EPA recommended monitoring for “total mercury” in screening fish tissue studies based on the conservative assumption that all mercury is present in fish tissue as methylmercury (USEPA, 2000a); therefore, total mercury was measured for the NRSA fish tissue studies. Mercury was detected in 100 % of the fillet samples from both studies at concentrations above the quantitation limit of 0.2 ng/g. Concentration ranges were similar for both studies, ranging from about 9 ng/g to 1070 ng/g for 2013–14 and from about 9 ng/g to 1340 ng/g for 2018–19. The median concentration was identical for both studies at 180 ng/g (Table 1). The median and other percentiles in Tables 1, A.9, and A.10 are statistical estimates for the U.S. river length sampled population (79,448 km in 2013–14 and 66,142 km in 2018–19), and the minimum and maximum concentrations are measured values. The 2013–14 and 2018–19 mean mercury concentrations were 228 ng/g and 244 ng/g, respectively, which are statistically valid means for the sampled population of U.S. rivers because mercury was found in every sample above the quantitation limit. A comparison of fish species that predominated during both surveys indicated that mercury levels were similar among Channel Catfish, Smallmouth Bass, and Largemouth Bass, as indicated by the lack of separation between their interquartile ranges (IQR) (Fig. 2).

#### 3.3.2. PCB detections and concentrations

EPA recommends reporting total PCB concentrations (calculated as the sum of the concentrations of the congeners or homologues) because the agency recognizes that the analysis of Aroclors (i.e., commercial PCB mixtures) alone does not adequately represent all of the potential sources of PCBs to aquatic environments or those PCBs found in fish tissue (USEPA, 2000b). Therefore, all fillet composite samples were analyzed for the full complement of 209 PCB congeners, either as individual congeners or groups of two to six coeluting congeners. The sum of all 209 PCB congeners (hereafter referred to as the total PCB concentration, or simply PCBs) was derived by assigning a value of zero to congeners that were not detected. Coelution patterns differed slightly across the two studies, yielding 162 congener measurements in the 2013–14 study and 159 congener measurements in the 2018–19 study. However, because the study results were assessed in terms of total PCB concentrations, these minor coelution differences did not affect the conclusions.

PCBs were detected in 100 % of the fillet samples from both surveys. Total PCB concentrations ranged from <1 ng/g (both surveys) to 4617 ng/g for 2013–14 and 1212 ng/g for 2018–19. Median concentrations were 12 ng/g and 9 ng/g for the 2013–14 and 2018–19 studies, respectively (Table 2). The median and all percentiles presented in Tables 2, A.11, and A.12 are statistical estimates for the sampled population of rivers (48,826 river km in 2013–14 and 66,142 river km in 2018–19) and the maximum values are measured concentrations. Comparisons for fish species that dominated in abundance showed that Channel Catfish (a top predator species in its adult life stage) had the highest maximum PCB concentrations for both surveys; however, there was no distinct difference or separation in the IQR of the three predominant species (Fig. 2).

#### 3.3.3. PFAS detections and concentrations

**Fig. 3.**
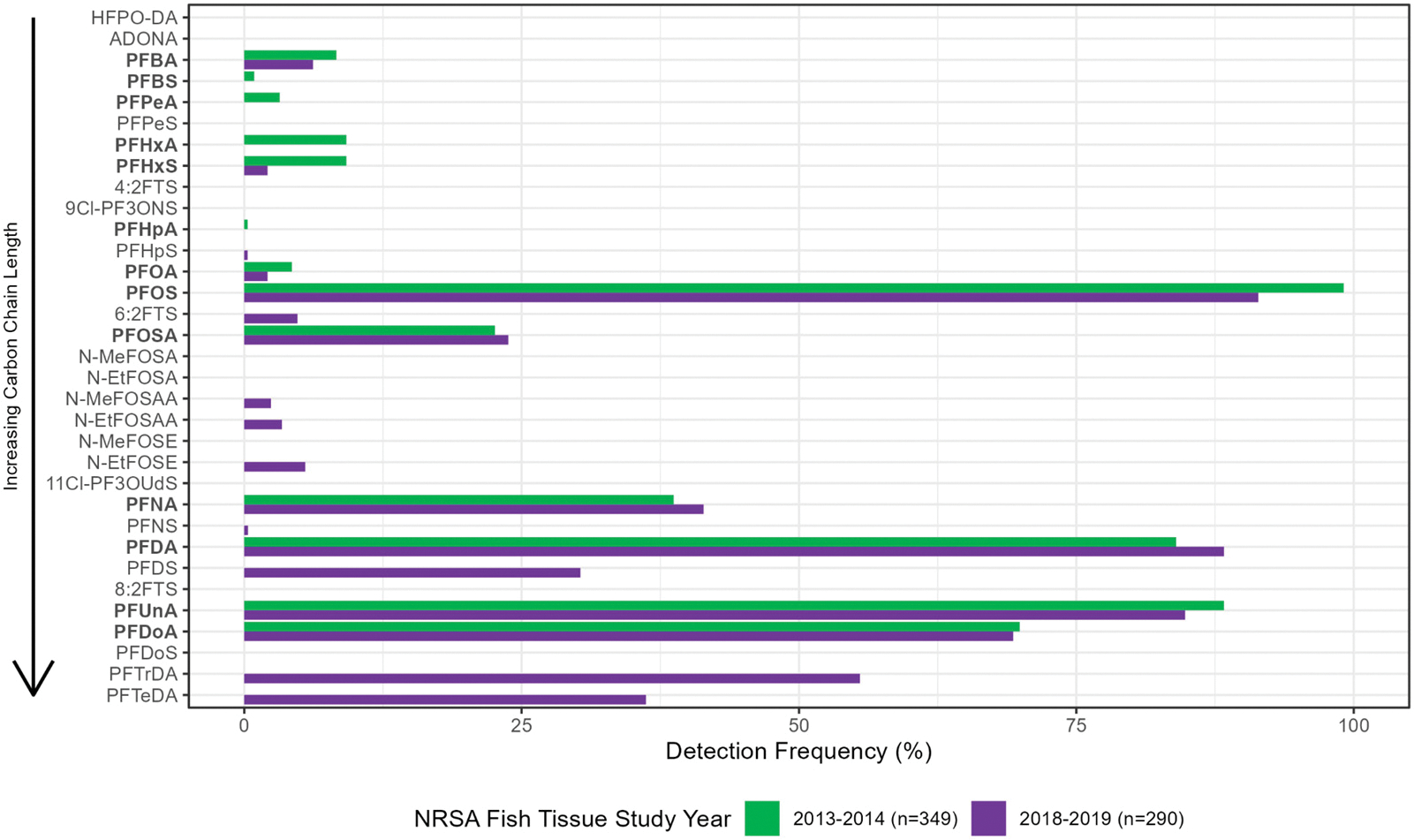
Detection frequency (% occurrence) of measured PFAS concentrations in fish fillet samples analyzed for the 2013–14 NRSA and 2018–19 NRSA fish tissue studies (PFAS in bold were analyzed during both studies).

PFAS were detected in 99.7 % and 95.2 % of the fillet samples from fish collected in 2013–14 and 2018–19, respectively. Only one sample from 2013 to 14 had no detections reported for all PFAS, and 14 samples from 2018 to 2019 had no PFAS detections. PFAS with seven or fewer carbon atoms (Table A.3) had low occurrences (Fig. 3), i.e., they were found in <1 % to 9 % of all 2013–14 samples and 0 % to 6 % of all 2018–19 samples (Table 3). For both studies, frequency of occurrence was dominated by PFOS (91 %–99 %), followed by three other longer-chain PFAS (85 %–88 % PFUnA, 84 %–88 % PFDA, and 69 %–70 % PFDoA). PFOS concentrations ranged from <1 ng/g (for both surveys) to 283 ng/g for 2013–14 and 131 ng/g for 2018–19, and the median concentration was 6 ng/g and 3 ng/g for 2013–14 and 2018–19, respectively (Table 3). The median and all other percentiles presented in Tables 3, A.13, and A.14 are statistical estimates for the sampled population of river length and the maximum value is a measured concentration. A comparison of fish species that predominated during both surveys indicated that PFOS levels were similar between Channel Catfish, Smallmouth Bass, and Largemouth Bass, as indicated by the lack of IQR separation (Fig. 2).

### 3.4. Contaminant probability distributions and fish tissue screening level exceedances

#### 3.4.1. Mercury

**Fig. 4.**
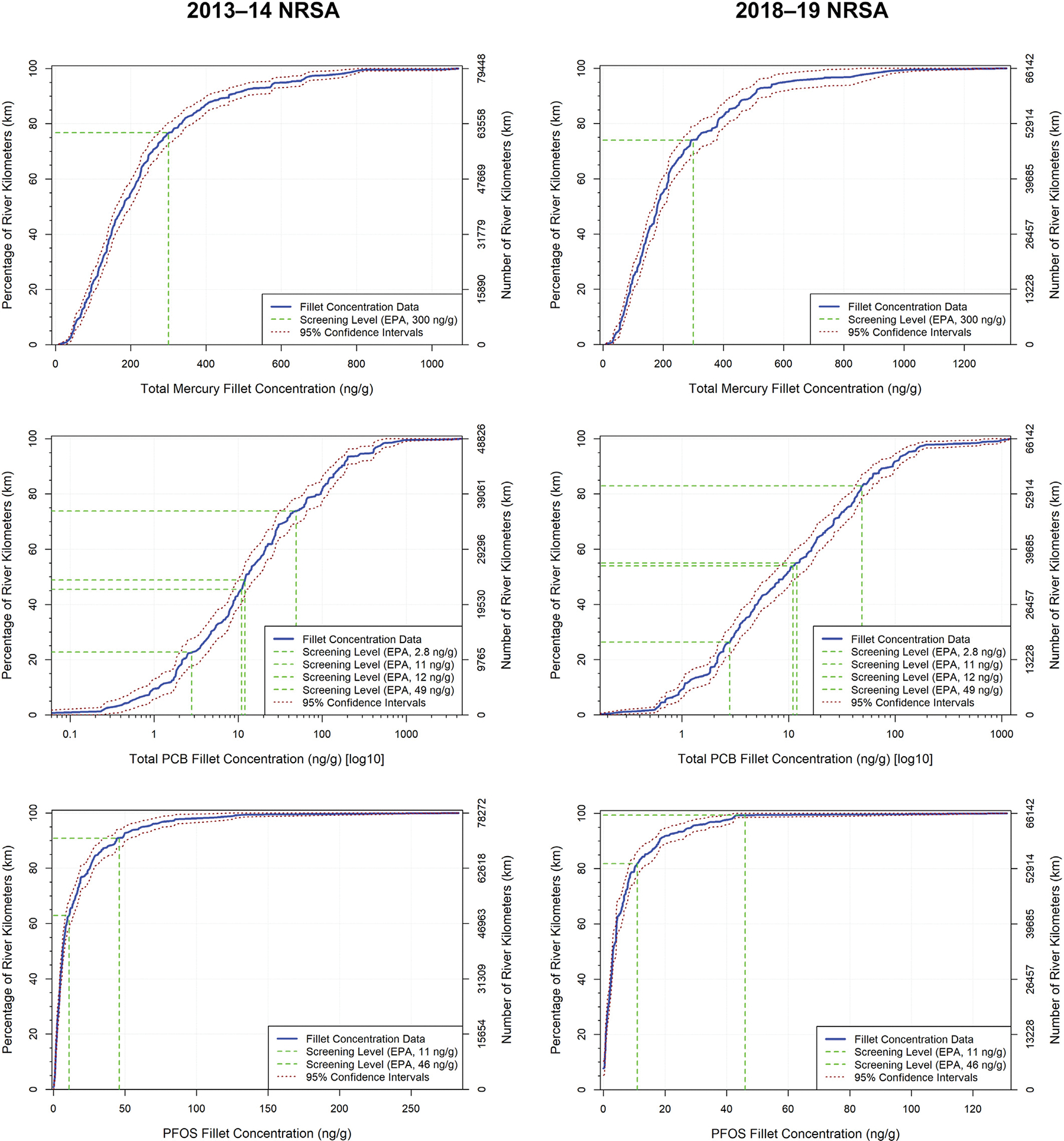
Cumulative distribution functions (CDFs) of total mercury, total PCB, and PFOS concentrations in fish fillet samples analyzed for the 2013–14 and 2018–19 NRSA fish tissue studies with fish tissue screening levels shown as vertical dotted line (log scale CDFs are provided for PCBs to increase screening level visibility).

**Fig. 5.**
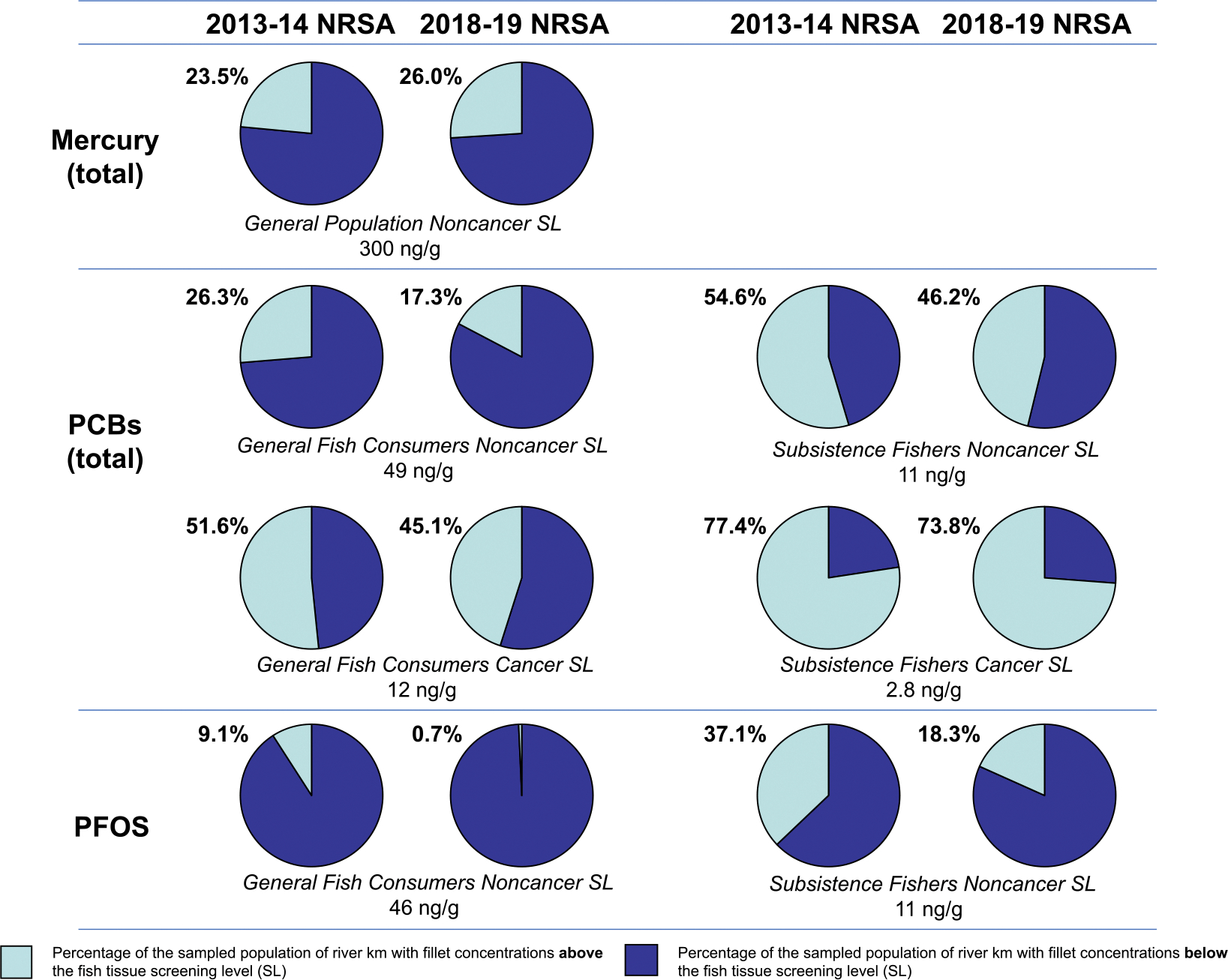
Percentage of the 2013–14 and 2018–19 NRSA sampled populations of U.S. river lengths containing fish with fillet concentrations that exceeded (light blue) fish tissue screening levels for total mercury, total PCBs, and PFOS.

Fish consumption can be a primary mercury exposure pathway for humans (Gandhi et al., 2016). Dellinger et al. (2013) emphasized that continued monitoring of mercury and other PBT chemicals in fish is critical, citing global use and long-range atmospheric transport of PBTs. Based on a mercury data compilation for the western U.S. and Canada, Eagles-Smith et al. (2016) concluded that mercury fish tissue contamination is widespread and heterogeneous in western North America, and Kamman et al. (2005) found that mercury levels in fish vary considerably by waterbody type and geographic area across North America. Fish tissue mercury concentrations in lotic waterbodies are the result of external loading more so than in situ streambed production, with less opportunity for biogeochemical reactions compared to lentic waters (Walters et al., 2010). Evaluations of the factors responsible for mercury accumulation in fish have typically focused on lentic habitats and are lacking for lotic systems (Mills et al., 2019). Walters et al. (2010) and Riva-Murray et al. (2020) agreed that little is known about spatial patterns or temporal changes in mercury in fish from rivers and streams. To provide a contemporary national perspective for U.S. rivers, fish tissue screening levels (Table A.1) were applied to 2013–14 and 2018–19 NRSA probability distributions of fish fillet contaminant levels, which allowed an estimation of the percentage of the sampled population of U.S. river length in km containing fish with fillet concentrations above a level protective of human health (Table 4). The screening level applied for mercury was the 300 ng/g USEPA tissue-based water quality criterion for methylmercury (USEPA, 2001b, 2010b), representing the concentration that should not be exceeded based on a total consumption-weighted rate of 0.0175 kg of fish/day (assuming a human adult body weight default value of 70 kg and an RfD of 0.0001 mg/kg-day). The mercury probability distributions (Fig. 4) showed that fish from 23.5 % (±1.9 %) (18,670 km) of the 2013–14 sampled population of 79,448 river km for mercury had fillet concentrations that exceeded the USEPA 300 ng/g fish tissue criterion. The exceedance percentage for mercury was similar in fillets from the 2018–19 study, with 26.0 % (±2.6 %) (17,197 km) of the sampled population of 66,142 river km containing fish with mercury fillet concentrations exceeding 300 ng/g (Fig. 5).

#### 3.4.2. PCBs

PCBs bioaccumulate in the aquatic environment, have slow degradation rates, and have high lipid solubilities; therefore, they remain detectable in fish today despite being banned from production and use in the U.S. for decades. In an assessment of persistent organic pollutants in fish from the mid-continental great rivers of the U.S. (i.e., the upper Mississippi, Missouri, and Ohio rivers), Blocksom et al. (2010) concluded that PCBs posed the greatest exposure risk to humans and were observed to a greater extent (at higher levels) than expected, considering their ban from production and use. For a national perspective on PCBs in U.S. river fish, screening levels for total PCBs (Table A.1) were applied to 2013–14 and 2018–19 NRSA fillet results to characterize human health risk and identify the percentage of the sampled population of river km that exceed PCB fish tissue screening levels (Table 4). EPA calculated PCB fish tissue screening levels to assess cancer and noncancer human health impacts to general fish consumers, using equations from USEPA (2000b). Inputs to those equations included an adult body weight of 80 kg (USEPA, 2011) and a nutrition-based fish consumption rate of 32.4 g/day for general fish consumers, consistent with the 2020–25 U.S. Department of Agriculture and Department of Health and Human Services’ Dietary Guidelines for Americans (USDA and USDHHS, 2020). This fish consumption rate better reflects the role and purpose of fish advisory programs because it is in line with the nutrition-based goals for dietary consumption (i.e., 32.4 g/day is equivalent to one 8-ounce meal of fish or shellfish per week). EPA also calculated PCB fish tissue screening levels for subsistence fishers, using an adult body weight of 80 kg and a fish consumption rate of 142 g/day, representing four to five 8-ounce meals per week (Table A.1) (USEPA, 2000c).

Four fish tissue screening levels (wet weight) are included in Tables A.1 and 4 for PCBs. The 2.8 ng/g PCB screening level is based on a cancer endpoint for subsistence fishers, and 12 ng/g is the PCB cancer screening level for general fish consumers. Both cancer health endpoints represent a 1 in 100,000 (10^−5^) risk level (USEPA, 2000b). The 11 ng/g and 49 ng/g screening levels are based on noncarcinogenic effects (e.g., reproductive effects in women and liver disease), and fish consumption rates of subsistence fishers and general fish consumers, respectively. Application of these PCB cancer and noncancer fish tissue screening levels to the total PCB fillet data identifies the number of U.S. river km and percentage of the sampled population of river km containing fish with total PCB fillet concentrations that are above each PCB fish tissue screening level.

The 2013–14 probability distributions for PCBs (Fig. 4) and general fish consumption rates (Table 4) show that fillet samples from fish in 51.6 % (±2.8 %) (25,194 km) of the PCB sampled population of 48,826 river km had total PCB concentrations that exceeded the 12 ng/g cancer screening level, and total PCB fillet concentrations in fish from 26.3 % (±2.3 %) (12,841 km) of this sampled population were over the 49 ng/g noncancer screening level (Fig. 5). Results based on subsistence fisher consumption rates from the 2013–14 survey (Figs. 4, 5, and Table 4) show that fillets from fish in 77.4 % (±2.6 %) (37,791 km) of the PCB sampled population of 48,826 river km had total PCB concentrations that exceeded the 2.8 ng/g cancer screening level, and total PCB fillet concentrations in fish from 54.6 % (±2.6 %) (26,659 km) of this sampled population were over the 11 ng/g noncancer screening level. The 2018–19 probability distributions for PCBs (Fig. 4) and general fish consumption rates (Table 4) show that fillet samples from fish in 45.1 % (±2.7 %) (29,830 km) of the sampled population of 66,142 river km had total PCB concentrations that exceeded the 12 ng/g cancer screening level, and total PCB fillet concentrations in fish from 17.3 % (±2.2 %) (11,443 km) of this sampled population were over the 49 ng/g noncancer screening level (Fig. 5). Results based on subsistence fisher consumption rates from the 2018–19 survey (Figs. 4, 5, and Table 4) show that fillets from fish in 73.8 % (±2.4 %) (48,813 km) of the sampled population of 66,142 river km had total PCB concentrations that exceeded the 2.8 ng/g cancer screening level, and total PCB fillet concentrations in fish from 46.2 % (±2.6 %) (30,558 km) of this sampled population were over the 11 ng/g noncancer screening level.

#### 3.4.3. PFAS

Goodrow et al. (2020) noted that PFAS bioaccumulation in fish may reach levels of human health concern even when concentrations in water are very low or even nondetectable. A published summary and analysis of PFAS monitoring results since 2005 by Houde et al. (2011) concluded that PFAS fish consumption advisories may be warranted considering their widespread presence and their elevated levels in particular locations. A global perspective and retrospective on PFAS (Kannan, 2011) showed that PFOS is the predominant perfluoroalkyl substance in humans and other biota. Goodrow et al. (2020) also found that PFOS was the most commonly detected PFAS in freshwater fish in an investigation of targeted waterbodies suspected to be impacted by PFAS in New Jersey, and they noted that PFOS levels in nearly all fish species were high enough to trigger fish consumption advisories. PFOS predominated in the 2013–14 and 2018–19 NRSA fish fillet samples as well. In 2008, the Minnesota Department of Health (MDH) was the first state agency to develop a PFOS RfD and include meal advice categories (based on PFOS levels in fish) as part of its statewide Fish Consumption Advisory Program (MDH, 2008).

In a 2016 health effects support document for PFOS (USEPA, 2016c), EPA published a derived RfD for PFOS of 0.00002 mg/kg-day. Fish tissue screening levels were derived for PFOS (Table A.1) using the same general approach as described above for PCBs, i.e., with calculations based on USEPA (2000b) guidance and the application of both nutrition-based fish consumption rates for general fish consumers and consumption rates for subsistence fishers. However, for PFOS screening level calculations, a 75-kg body weight from EPA’s 2011 Exposure Factors Handbook (USEPA, 2011) was used because it is associated with the population that was the basis for the RfD derivation (i.e., the RfD was based on developmental effects, so the body weight is for pregnant or lactating women).

Application of fish tissue screening levels to the national probabilistic results for PFOS (Figs. 4, 5, and Table 4) indicates that in 2013–14, 9.1 % (±1.6 %) (7123 km) of the PFOS sampled population of 78,272 river km had fish with PFOS fillet tissue concentrations that exceeded the 46 ng/g noncancer screening level for general fish consumers, and PFOS fillet tissue concentrations in fish from 37.1 % (±2.5 %) (29,039 km) of this sampled population exceeded the 11 ng/g noncancer screening level for subsistence fishers. In 2018–19, fish in 0.7 % (±0.4 %) (463 km) of the sampled population of 66,142 river km had PFOS fillet tissue concentrations that exceeded the 46 ng/g noncancer screening level for general fish consumers, and PFOS fillet tissue concentrations in fish from 18.3 % (±2.2 %) (12,104 km) of this sampled population exceeded the 11 ng/g noncancer screening level for subsistence fishers (Figs. 4, 5, and Table 4).

### 3.5. Estimates of temporal changes

Contaminant levels change over time due to chemical degradation, contaminant remediation, and regulatory controls. Riva-Murray et al. (2020) noted variable temporal changes in mercury fish tissue concentrations among and within rivers in New York State. They observed some declines over time, locations with no significant change over time, and some increases over time. In their summary of mercury trends in fish from U.S. lakes and rivers, Chalmers et al. (2011) concluded that they anticipated that mercury concentrations in fish will decrease in response to decreases in atmospheric mercury loading, but the magnitude and timing of that response is uncertain. In mid-continental great rivers of the U.S., Blocksom et al. (2010) noted that they found PCBs in fish to a greater extent and in higher-than-expected levels, considering that they have been banned from manufacture or use for decades. In their assessment of 2005–13 PFAS fish tissue data from the Upper Mississippi River, Newsted et al. (2017) found that there was a consistent downward trend in PFOS concentrations for most species; whereas an assessment of PFAS in Delaware River fish by MacGillivray (2021) found that changes in PFAS concentrations from 2004 to 2018 were variable (i.e., decreases were noted for some PFAS but not others). The potential for change emphasizes the importance of documenting baseline contaminant levels, as well as a need for surveillance monitoring over time.

In addition to addressing the primary goal of estimating current contaminant levels in U.S. rivers, the NRSA fish tissue studies also included a design component to address the secondary objective of estimating change in fish contaminants over time. Change estimates for the 5-year timespan are summarized in Table 5. Estimate comparisons indicated no significant change in total mercury average levels or fish tissue screening level exceedances between the two studies (at the alpha level of 0.05). Fish tissue mercury screening level exceedance change estimates for the sampled population of river km showed an increase of 2.5 %, but that change was not significant at the alpha level of 0.05 (Table 5). Mercury results were also available from 2008 to 2009 NRSA fish fillet samples, allowing a 10-year change estimate when compared to 2018–19 results (i.e., a comparison that was not possible for PFAS or PCBs because the 2008–09 study included only urban samples for PFAS analysis, and only 21 PCB congeners were analyzed for the 2008–09 study versus 209 congeners for the 2013–14 and 2018–19 studies). Fish tissue mercury screening level exceedances for the sampled population of river km showed an increase of 4.6 % for the 10-year time span, but that change was not significant at the alpha level of 0.05. A comparison of PCB results between the 2013–14 and 2018–19 studies indicated that there were significant decreases in exceedance percentages for the noncancer fish tissue screening levels for both general fish consumers (49 ng/g screening level) and subsistence fishers (11 ng/g screening level), with a 9.0 % and 8.4 % decrease, respectively. Although PCB cancer screening level exceedances decreased by 6.4 % for general fish consumers (12 ng/g screening level) and by 3.6 % for subsistence fishers (2.8 ng/g screening level), those changes were not significant (at the alpha level of 0.05). Fish tissue screening level exceedances for PFOS decreased by 8.4 % for general fish consumers (using a 46 ng/g noncancer screening level) and by 18.9 % for subsistence fishers (using an 11 ng/g noncancer screening level), and those decreases were significant at the alpha level of 0.05.

These surveys evaluate risks from human consumption of fish from U.S. rivers based on contaminant results from fish that were available from random sampling locations during specific sampling periods at 5- or 10-year intervals. The objective was to determine the proportion of the nation’s rivers that contain fish with concentrations of the target contaminants that exceed human health fish tissue screening levels. This contrasts with the approach of periodically collecting a specific species of fish at the same sampling locations (i.e., targeted fish sampling) to serve as contaminant biomarkers. Change analyses indicated statistically significant population-level changes for some target analytes between the 2013–14 and 2018–19 studies. These population-level changes represent a change in human health risk associated with eating the species and sizes of fish available for consumption during the particular survey years and may be the result of differences in fish species and/or sizes between the two surveys rather than changes in contaminant levels in U.S. rivers. Regardless of these differences, each survey met the objective of obtaining fish that are commonly caught and consumed by recreational and subsistence fishers. Comparing results from a single species common to both studies may add perspective on changes in contaminant levels in U.S. rivers. For example, Fig. 2 compares Channel Catfish to Channel Catfish, Smallmouth Bass to Smallmouth Bass, and Largemouth Bass to Largemouth Bass between survey years. Mercury, PCB, and PFOS results for those predominant species showed overlapping IQR when comparing 2013–14 and 2018–19 results. That indicates that there were little to no changes within species between the two surveys despite significant target population-level changes in fillet tissue screening level exceedances for PCBs and PFOS using the full complement of species represented in the fish samples.

## 4. Conclusions

Fish are an important source of protein and essential fatty acids that provide nutritional human health benefits, but they may also be pathways for exposure to mercury and other contaminants. Fish contaminant studies focus on human health risks and are not intended to discourage consumption of fish, but rather to offer recommendations on safe consumption levels to protect human health. Most historical fish contamination assessments have focused on waters that were known to be impacted by a particular chemical or group of chemicals, and past fish tissue studies in rivers are either limited in scale or do not employ random site selection to allow extrapolation of results to locations that were not sampled. In order to estimate the national extent of contamination in river fish in the U.S. during the 2013–14 and 2018–19 NRSA studies, a probability-based approach (in this case, a GRTS survey design) was essential. The survey design incorporated statistical techniques to ensure that sampling sites were randomly selected and well distributed throughout the conterminous U.S. State agencies in the U.S. have recognized the utility of the probabilistic design and have incorporated it into their monitoring programs to provide statistically defensible data to characterize the relative risks associated with the consumption of fish. This survey design could be applied on smaller and larger spatial scales in streams, rivers, lakes, and coastal waters if the target population being sampled is clearly defined and the sample selection criteria are met. The resulting cumulative national data distributions from the NRSA studies, combined with the application of fish tissue screening levels for human health, allowed an estimation of the number or percent of river km in the sampled population of U.S. rivers that contain fish with fillet tissue concentrations above a level protective of human health. These fish fillet tissue study results provide valuable information for characterizing relative risks associated with fish consumption and represent a baseline point in time that may be useful for evaluating the effectiveness of ongoing or future contaminant controls, remedial actions, and management initiatives. For future research it is important to consider that, as the science and understanding of contaminant toxicology evolves, reference dose values may change, which in turn will require revisions to fish tissue screening levels for the interpretation of relative human health risks.

## Supplementary Material

Supplement1

## Figures and Tables

**Table 1:** Percentiles, minimum detected and maximum measured concentrations (ng/g, wet weight), and frequency of occurrence for total mercury detected in NRSA fish fillet tissue samples.

Chemical	Year	Number of detections	MDL^a^	Minimum	Percentile^b^				Maximum	Frequency of occurrence^c^
					25th	50th	75th	90th		

Mercury (total)	2013–14	353	0.06	8.61	111	180	288	461	1070	100 %
	2018–19	290	0.09	9.44	101	180	315	495	1340	100 %

a MDL = Method Detection Limit in ng/g, wet weight.

b Percentile concentrations are statistical estimates for the sampled population of river length determined for each study (a length of 79,448 km for the 2013–14 study and 66,142 km for the 2018–19 study); minimum and maximum concentrations are measured values at 353 sites in 2013–14 and 290 sites in 2018–19.

c Percent frequency of occurrence values are based on 353 and 290 possible detections in 2013–14 and 2018–19, respectively.

**Table 2:** Percentiles, minimum and maximum calculated concentrations (ng/g, wet weight), and frequency of occurrence for total PCBs detected in NRSA fish fillet tissue samples.

Chemical	Year	Number of detections	Minimum	Percentile^a^				Maximum	Frequency of occurrence^b^
				25th	50th	75th	90th		

Total PCBs	2013–14	223	0.060	3.58	12.3	57.7	171	4617	100 %
(Sum of 209 congeners)	2018–19	290	0.171	2.52	9.04	35.3	94.8	1212	100 %

a Percentile concentrations are statistical estimates for the sampled population of river length determined for each study (a length of 48,826 km for the 2013–14 study and 66,142 km for the 2018–19 study); minimum and maximum concentrations are the minimum and maximum calculated values at 223 sites in 2013–14 and 290 sites in 2018–19.

b Percent frequency of occurrence values are based on 223 and 290 possible detections in 2013–14 and 2018–19, respectively.

**Table 3:** Percentiles, minimum detected and maximum measured concentrations (ng/g, wet weight), and frequency of occurrence for the 13 PFAS (listed in order of increasing carbon chain length) common to both the 2013–14 and 2018–19 studies and detected in NRSA fish fillet tissue samples.

Chemical^a^	Year	Number of detections	MDL^b^	Minimum	Percentile^c^				Maximum	Frequency of occurrence^d^
					25th	50th	75th	90th		

PFBA	2013–14	29	0.100	0.111	<MDL	<MDL	<MDL	<MDL	48.1	8.3 %
PFBS		3	0.100	0.148	<MDL	<MDL	<MDL	<MDL	0.571	0.9 %
PFPeA		11	0.069	0.360	<MDL	<MDL	<MDL	<MDL	0.884	3.2 %
PFHxA		32	0.052	0.115	<MDL	<MDL	<MDL	<MDL	1.44	9.2 %
PFHxS		32	0.066	0.121	<MDL	<MDL	<MDL	0.0252	0.980	9.2 %
PFHpA		1	0.060	0.660	<MDL	<MDL	<MDL	<MDL	0.660	0.3 %
PFOA		15	0.110	0.111	<MDL	<MDL	<MDL	<MDL	0.271	4.3 %
PFOS		346	0.077	0.155	2.85	6.49	18.8	43.9	283	99.1 %
PFOSA		79	0.071	0.116	<MDL	<MDL	<MDL	0.369	35.0	22.6 %
PFNA		135	0.043	0.100	<MDL	<MDL	0.267	0.660	1.91	38.7 %
PFDA		293	0.073	0.115	0.226	0.580	1.16	2.12	18.0	84.0 %
PFUnA		308	0.074	0.133	0.317	0.621	1.37	2.56	53.9	88.3 %
PFDoA		244	0.059	0.100	<MDL	0.308	0.742	1.90	99.5	69.9 %
PFBA	2018–19	18	0.551	0.517	<MDL	<MDL	<MDL	<MDL	0.806	6.2 %
PFBS		0	0.097	0.000	<MDL	<MDL	<MDL	<MDL	0.00	0.0 %
PFPeA		0	0.192	0.000	<MDL	<MDL	<MDL	<MDL	0.00	0.0 %
PFHxA		0	0.203	0.000	<MDL	<MDL	<MDL	<MDL	0.00	0.0 %
PFHxS		6	0.153	0.194	<MDL	<MDL	<MDL	<MDL	0.611	2.1 %
PFHpA		0	0.170	0.000	<MDL	<MDL	<MDL	<MDL	0.00	0.0 %
PFOA		6	0.162	0.161	<MDL	<MDL	<MDL	<MDL	0.354	2.1 %
PFOS		265	0.354	0.353	1.15	3.07	7.97	18.2	131	91.4 %
PFOSA		69	0.152	0.149	<MDL	<MDL	<MDL	0.250	2.87	23.8 %
PFNA		120	0.129	0.122	<MDL	<MDL	0.176	0.273	1.44	41.4 %
PFDA		256	0.116	0.115	0.200	0.332	0.655	1.23	29.8	88.3 %
PFUnA		246	0.151	0.143	0.237	0.516	0.939	1.52	105	84.8 %
PFDoA		201	0.156	0.155	<MDL	0.277	0.533	1.30	140	69.3 %

a The 13 PFAS analyzed in both the 2013–14 and 2018–19 NRSA studies are a subset of the 33 PFAS analyzed for the 2018–19 study (detailed in Table A.13).

b MDL = Method Detection Limit in ng/g, wet weight, based on the nominal sample mass analyzed. Because some samples were analyzed using a slightly larger mass, some of the minimum values in this table may be slightly below the nominal MDL values shown.

c Percentile concentrations are statistical estimates for the sampled population of river length determined for each study (a length of 78,272 km for 2013–14 and 66,142 km for 2018–19); minimum and maximum concentrations are measured values at 349 sites in 2013–14 and 290 sites in 2018–19.

d Percent frequency of occurrence values are based on 349 and 290 possible detections in 2013–14 and 2018–19, respectively.

**Table 4:** Fish tissue screening levels for assessing cancer and noncancer human health risks applied to 2013–14 and 2018–19 NRSA results.

Chemical	Category for assessing human health risks	Fish tissue screening level (ng/g, wet weight)	Fish consumption rate	Percent of sampled population of river kilometers with fish having fillet concentrations exceeding screening level (and associated standard error)	
				2013 – 2014^a^	2018 – 2019^b^

Mercury (total)	Noncancer screening level (general population)	300 ng/g	17.5 g/day^c^	23.5% (±1.9)	26.0% (±2.6)
PCBs (total)	Cancer screening level (subsistence fishers)	2.8 ng/g	142 g/day^d^	77.4% (±2.6)	73.8% (±2.4)
	Noncancer screening level (subsistence fishers)	11 ng/g	142 g/day^d^	54.6% (±2.6)	46.2% (±2.6)
	Cancer screening level (general fish consumers)	12 ng/g	32.4 g/day^e^	51.6% (±2.8)	45.1 % (±2.7)
	Noncancer screening level (general fish consumers)	49 ng/g	32.4 g/day^e^	26.3% (±2.3)	17.3% (±2.2)
PFOS	Noncancer screening level (subsistence fishers)	11 ng/g	142 g/day^d^	37.1 % (±2.5)	18.3% (±2.2)
	Noncancer screening level (general fish consumers)	46 ng/g	32.4 g/day^e^	9.1 % (±1.6)	0.7% (±0.4)

a The sampled populations of river lengths determined for 2013–14 are 79,448 km for total mercury, 48,826 km for total PCBs, and 78,272 km for PFOS because different numbers of fillet samples were analyzed for each chemical or chemical group.

b The sampled population of river length determined for 2018–19 is 66,142 km for all analytes.

c Fish consumption rate for adult general population that was used to derive the methylmercury water quality criterion (based on USEPA, 2001b).

d Fish consumption rate for subsistence fishers (based on USEPA, 2000c).

e Fish consumption rate for general fish consumers; one 8-oz serving per week (based on guidelines described in USDA and USDHHS, 2020).

**Table 5:** Estimates of temporal changes between NRSA fish fillet tissue probabilistic survey results for total mercury, total PCBs, and PFOS.

		2013–14^a^	2018–19^b^	Change estimates	

Mercury (total)	Samples (n)	353	290		
	Noncancer – general population	23.5	26.0	↑	2.5 %
	300 ng/g screening level exceedance (%)				
PCBs (total)	Samples (n)	223	290		
	Cancer – subsistence fishers	77.4	73.8	↓	3.6 %
	2.8 ng/g screening level exceedance (%)				
	Noncancer – subsistence fishers	54.6	46.2	↓	8.4 %*
	11 ng/g screening level exceedance (%)				
	Cancer – general fish consumers	51.6	45.1	↓	6.4 %
	12 ng/g screening level exceedance (%)				
	Noncancer – general fish consumers	26.3	17.3	↓	9.0 %*
	49 ng/g screening level exceedance (%)				
PFOS	Samples (n)	349	290		
	Noncancer – subsistence fishers	37.1	18.3	↓	18.9 %*
	11 ng/g screening level exceedance (%)				
	Noncancer – general fish consumers	9.1	0.7	↓	8.4 %*
	46 ng/g screening level exceedance (%)				

↓ Indicates a decrease in concentration or screening level exceedance percentage; ↑ indicates an increase in concentration or screening level exceedance percentage.

a The sampled populations of river lengths determined for the 2013–14 study are 79,448 km for total mercury, 48,826 km for total PCBs, and 78,272 km for PFOS because different numbers of fillet samples were analyzed for each chemical or chemical group.

b The sampled population of river length determined for 2018–19 is 66,142 km for all analytes.

* Significant difference at the alpha level of 0.05.

## Data Availability

Data will be made available on request.

## HIGHLIGHTS

- Fish contamination characterized for up to 79,448 United States river kilometers.
- Mercury and polychlorinated biphenyls detected in all fish fillets and rivers.
- Per- and polyfluoroalkyl substances detected in 95.2–99.7 % of fish samples annually.
- Human health fish tissue screening levels exceeded for all target chemicals.
