# Supplementary material for: Contaminants in fish from U.S. rivers: Probability-based national assessments
Source: Sci Total Environ. Author manuscript; Available in PMC 2024 Feb 25. (PMC9948096; doi:10.1016/j.scitotenv.2022.160557)
Supplement: Supplement1 [file NIHMS1872376-supplement-Supplement1.docx]

**Appendix A: Supplementary Material**

**Fish tissue screening levels, target chemical lists for PCBs and PFAS, fish species list,**

**analytical QC data summaries, and analytical and statistical results**

**Table A.1. Fish tissue screening levels for assessing cancer and noncancer human health risks applied to NRSA 2013–14 and 2018–19 results.**

| **Chemical** | **Category for assessing human health risks** | **Fish tissue screening level (wet weight)** | **Fish consumption basis** | **Fish tissue screening level source** |
| --- | --- | --- | --- | --- |
| Mercury (total) | Noncancer screening level (general population) | 300 ng/g | 17.5 g/day^a^ | USEPA tissue-based methylmercury water quality criterion (USEPA, 2001) |
| PCBs (total) | Cancer screening level (subsistence fishers) | 2.8 ng/g | 142 g/day^b^ | Based on equations described in Guidance for Assessing Chemical Contaminant Data for Use in Fish Advisories: Vol. 2 (USEPA, 2000b) and the national default value for an average adult body weight in the Exposure Factors Handbook (USEPA, 2011) |
|  | Noncancer screening level (subsistence fishers) | 11 ng/g | 142 g/day^b^ |  |
|  | Cancer screening level (general fish consumers) | 12 ng/g | 32.4 g/day^c^ |  |
|  | Noncancer screening level (general fish consumers) | 49 ng/g | 32.4 g/day^c^ |  |
| PFOS | Noncancer screening level (subsistence fishers) | 11 ng/g | 142 g/day^b^ | Based on equations described in Guidance for Assessing Chemical Contaminant Data for Use in Fish Advisories: Vol. 2 (USEPA, 2000b) and the human health RfD in USEPA’s Health Effects Support Document for PFOS (USEPA, 2016c) and the national default value for a pregnant woman body weight in the Exposure Factors Handbook (USEPA, 2011) |
|  | Noncancer screening level (general fish consumers) | 46 ng/g | 32.4 g/day^c^ |  |

^a^ Fish consumption rate for adult general population used to derive the methylmercury water quality criterion (based on USEPA, 2001b).

^b^ Fish consumption rate for subsistence fishers (based on USEPA, 2000c).

^c^ Fish consumption rate for general fish consumers; one 8-oz serving per week (based on guidelines described in USDA and USDHHS, 2020).

| **Table A.2. PCB congeners and CAS Registry Numbers analyzed for the 2013–14 and 2018–19 National Rivers and Streams Assessment (NRSA) fish tissue studies.** | | | | | | | |
| --- | --- | --- | --- | --- | --- | --- | --- |
| **Congener^a^** | **CAS Number^b^** | **Congener^a^** | **CAS Number^b^** | **Congener^a^** | **CAS Number^b^** | **Congener^a^** | **CAS Number^b^** |
| PCB-1 | 2051-60-7 | PCB-32 | 38444-77-8 | PCB-63 | 74472-34-7 | PCB-94 | 73575-55-0 |
| PCB-2 | 2051-61-8 | PCB-33 | 38444-86-9 | PCB-64 | 52663-58-8 | PCB-95 | 38379-99-6 |
| PCB-3 | 2051-62-9 | PCB-34 | 37680-68-5 | PCB-65 | 33284-54-7 | PCB-96 | 73575-54-9 |
| PCB-4 | 13029-08-8 | PCB-35 | 37680-69-6 | PCB-66 | 32598-10-0 | PCB-97 | 41464-51-1 |
| PCB-5 | 16605-91-7 | PCB-36 | 38444-87-0 | PCB-67 | 73575-53-8 | PCB-98 | 60233-25-2 |
| PCB-6 | 25569-80-6 | PCB-37 | 38444-90-5 | PCB-68 | 73575-52-7 | PCB-99 | 38380-01-7 |
| PCB-7 | 33284-50-3 | PCB-38 | 53555-66-1 | PCB-69 | 60233-24-1 | PCB-100 | 39485-83-1 |
| PCB-8 | 34883-43-7 | PCB-39 | 38444-88-1 | PCB-70 | 32598-11-1 | PCB-101 | 37680-73-2 |
| PCB-9 | 34883-39-1 | PCB-40 | 38444-93-8 | PCB-71 | 41464-46-4 | PCB-102 | 68194-06-9 |
| PCB-10 | 33146-45-1 | PCB-41 | 52663-59-9 | PCB-72 | 41464-42-0 | PCB-103 | 60145-21-3 |
| PCB-11 | 2050-67-1 | PCB-42 | 36559-22-5 | PCB-73 | 74338-23-1 | PCB-104 | 56558-16-8 |
| PCB-12 | 2974-92-7 | PCB-43 | 70362-46-8 | PCB-74 | 32690-93-0 | PCB-105 | 32598-14-4 |
| PCB-13 | 2974-90-5 | PCB-44 | 41464-39-5 | PCB-75 | 32598-12-2 | PCB-106 | 70424-69-0 |
| PCB-14 | 34883-41-5 | PCB-45 | 70362-45-7 | PCB-76 | 70362-48-0 | PCB-107 | 70424-68-9 |
| PCB-15 | 2050-68-2 | PCB-46 | 41464-47-5 | PCB-77 | 32598-13-3 | PCB-108 | 70362-41-3 |
| PCB-16 | 38444-78-9 | PCB-47 | 2437-79-8 | PCB-78 | 70362-49-1 | PCB-109 | 74472-35-8 |
| PCB-17 | 37680-66-3 | PCB-48 | 70362-47-9 | PCB-79 | 41464-48-6 | PCB-110 | 38380-03-9 |
| PCB-18 | 37680-65-2 | PCB-49 | 41464-40-8 | PCB-80 | 33284-52-5 | PCB-111 | 39635-32-0 |
| PCB-19 | 38444-73-4 | PCB-50 | 62796-65-0 | PCB-81 | 70362-50-4 | PCB-112 | 74472-36-9 |
| PCB-20 | 38444-84-7 | PCB-51 | 68194-04-7 | PCB-82 | 52663-62-4 | PCB-113 | 68194-10-5 |
| PCB-21 | 55702-46-0 | PCB-52 | 35693-99-3 | PCB-83 | 60145-20-2 | PCB-114 | 74472-37-0 |
| PCB-22 | 38444-85-8 | PCB-53 | 41464-41-9 | PCB-84 | 52663-60-2 | PCB-115 | 74472-38-1 |
| PCB-23 | 55720-44-0 | PCB-54 | 15968-05-5 | PCB-85 | 65510-45-4 | PCB-116 | 18259-05-7 |
| PCB-24 | 55702-45-9 | PCB-55 | 74338-24-2 | PCB-86 | 55312-69-1 | PCB-117 | 68194-11-6 |
| PCB-25 | 55712-37-3 | PCB-56 | 41464-43-1 | PCB-87 | 38380-02-8 | PCB-118 | 31508-00-6 |
| PCB-26 | 38444-81-4 | PCB-57 | 70424-67-8 | PCB-88 | 55215-17-3 | PCB-119 | 56558-17-9 |
| PCB-27 | 38444-76-7 | PCB-58 | 41464-49-7 | PCB-89 | 73575-57-2 | PCB-120 | 68194-12-7 |
| PCB-28 | 7012-37-5 | PCB-59 | 74472-33-6 | PCB-90 | 68194-07-0 | PCB-121 | 56558-18-0 |
| PCB-29 | 15862-07-4 | PCB-60 | 33025-41-1 | PCB-91 | 68194-05-8 | PCB-122 | 76842-07-4 |
| PCB-30 | 35693-92-6 | PCB-61 | 33284-53-6 | PCB-92 | 52663-61-3 | PCB-123 | 65510-44-3 |
| PCB-31 | 16606-02-3 | PCB-62 | 54230-22-7 | PCB-93 | 73575-56-1 | PCB-124 | 70424-70-3 |

| PCB-125 | 74472-39-2 | PCB-147 | 68194-13-8 | PCB-169 | 32774-16-6 | PCB-191 | 74472-50-7 |  |  |  |  |  |  |  |
| --- | --- | --- | --- | --- | --- | --- | --- | --- | --- | --- | --- | --- | --- | --- |
| PCB-126 | 57465-28-8 | PCB-148 | 74472-41-6 | PCB-170 | 35065-30-6 | PCB-192 | 74472-51-8 |  |  |  |  |  |  |  |
| PCB-127 | 39635-33-1 | PCB-149 | 38380-04-0 | PCB-171 | 52663-71-5 | PCB-193 | 69782-91-8 |  |  |  |  |  |  |  |
| PCB-128 | 38380-07-3 | PCB-150 | 68194-08-1 | PCB-172 | 52663-74-8 | PCB-194 | 35694-08-7 |  |  |  |  |  |  |  |
| PCB-129 | 55215-18-4 | PCB-151 | 52663-63-5 | PCB-173 | 68194-16-1 | PCB-195 | 52663-78-2 |  |  |  |  |  |  |  |
| PCB-130 | 52663-66-8 | PCB-152 | 68194-09-2 | PCB-174 | 38411-25-5 | PCB-196 | 42740-50-1 |  |  |  |  |  |  |  |
| PCB-131 | 61798-70-7 | PCB-153 | 35065-27-1 | PCB-175 | 40186-70-7 | PCB-197 | 33091-17-7 |  |  |  |  |  |  |  |
| PCB-132 | 38380-05-1 | PCB-154 | 60145-22-4 | PCB-176 | 52663-65-7 | PCB-198 | 68194-17-2 |  |  |  |  |  |  |  |
| PCB-133 | 35694-04-3 | PCB-155 | 33979-03-2 | PCB-177 | 52663-70-4 | PCB-199 | 52663-75-9 |  |  |  |  |  |  |  |
| PCB-134 | 52704-70-8 | PCB-156 | 38380-08-4 | PCB-178 | 52663-67-9 | PCB-200 | 52663-73-7 |  |  |  |  |  |  |  |
| PCB-135 | 52744-13-5 | PCB-157 | 69782-90-7 | PCB-179 | 52663-64-6 | PCB-201 | 40186-71-8 |  |  |  |  |  |  |  |
| PCB-136 | 38411-22-2 | PCB-158 | 74472-42-7 | PCB-180 | 35065-29-3 | PCB-202 | 2136-99-4 |  |  |  |  |  |  |  |
| PCB-137 | 35694-06-5 | PCB-159 | 39635-35-3 | PCB-181 | 74472-47-2 | PCB-203 | 52663-76-0 |  |  |  |  |  |  |  |
| PCB-138 | 35065-28-2 | PCB-160 | 41411-62-5 | PCB-182 | 60145-23-5 | PCB-204 | 74472-52-9 |  |  |  |  |  |  |  |
| PCB-139 | 56030-56-9 | PCB-161 | 74472-43-8 | PCB-183 | 52663-69-1 | PCB-205 | 74472-53-0 |  |  |  |  |  |  |  |
| PCB-140 | 59291-64-4 | PCB-162 | 39635-34-2 | PCB-184 | 74472-48-3 | PCB-206 | 40186-72-9 |  |  |  |  |  |  |  |
| PCB-141 | 52712-04-6 | PCB-163 | 74472-44-9 | PCB-185 | 52712-05-7 | PCB-207 | 52663-79-3 |  |  |  |  |  |  |  |
| PCB-142 | 41411-61-4 | PCB-164 | 74472-45-0 | PCB-186 | 74472-49-4 | PCB-208 | 52663-77-1 |  |  |  |  |  |  |  |
| PCB-143 | 68194-15-0 | PCB-165 | 74472-46-1 | PCB-187 | 52663-68-0 | PCB-209 | 2051-24-3 |  |  |  |  |  |  |  |
| PCB-144 | 68194-14-9 | PCB-166 | 41411-63-6 | PCB-188 | 74487-85-7 |  |  |  |  |  |  |  |  |  |
| PCB-145 | 74472-40-5 | PCB-167 | 52663-72-6 | PCB-189 | 39635-31-9 |  |  |  |  |  |  |  |  |  |
| PCB-146 | 51908-16-8 | PCB-168 | 59291-65-5 | PCB-190 | 41411-64-7 |  |  |  |  |  |  |  |  |  |
| ^a^ Congeners that were separated from one another and those that coeluted in each study varied slightly between the two studies and are shown in Tables A.10 and A.11.  ^b^ CAS Number = Chemical Abstracts Service Registry Number | | | | | | | |  |  |  |  |  |  |  |

| **Table A.3. Names, abbreviations, CAS registry numbers, and carbon chain length for target PFAS analyzed for the 2013–14 and 2018–19 NRSA fish tissue studies.** | | | | |
| --- | --- | --- | --- | --- |
| **Target analyte** | **Abbreviation** | **Study** | **CAS Number^a^** | **Carbon chain length** |
| **Perfluoroalkyl carboxylic acids** | | | | |
| Perfluorobutanoic acid | PFBA | 2013–14 and 2018–19 | 375-22-4 | 4 |
| Perfluoropentanoic acid | PFPeA | 2013–14 and 2018–19 | 2706-90-3 | 5 |
| Perfluorohexanoic acid | PFHxA | 2013–14 and 2018–19 | 307-24-4 | 6 |
| Perfluoroheptanoic acid | PFHpA | 2013–14 and 2018–19 | 375-85-9 | 7 |
| Perfluorooctanoic acid | PFOA | 2013–14 and 2018–19 | 335-67-1 | 8 |
| Perfluorononanoic acid | PFNA | 2013–14 and 2018–19 | 375-95-1 | 9 |
| Perfluorodecanoic acid | PFDA | 2013–14 and 2018–19 | 335-76-2 | 10 |
| Perfluoroundecanoic acid | PFUnA | 2013–14 and 2018–19 | 2058-94-8 | 12 |
| Perfluorododecanoic acid | PFDoA | 2013–14 and 2018–19 | 307-55-1 | 12 |
| Perfluorotridecanoic acid | PFTrDA | 2018–19 | 72629-94-8 | 13 |
| Perfluorotetradecanoic acid | PFTeDA | 2018–19 | 376-06-7 | 14 |
| **Perfluoroalkyl sulfonic acids** | | | | |
| Perfluorobutanesulfonic acid | PFBS | 2013–14 and 2018–19 | 375-73-5 | 4 |
| Perfluoropentansulfonic acid | PFPeS | 2018–19 | 2706-91-4 | 5 |
| Perfluorohexanesulfonic acid | PFHxS | 2013–14 and 2018–19 | 355-46-4 | 6 |
| Perfluoroheptanesulfonic acid | PFHpS | 2018–19 | 375-92-8 | 7 |
| Perfluorooctanesulfonic acid | PFOS | 2013–14 and 2018–19 | 1763-23-1 | 8 |
| Perfluorononanesulfonic acid | PFNS | 2018–19 | 68259-12-1 | 9 |
| Perfluorodecanesulfonic acid | PFDS | 2018–19 | 335-77-3 | 10 |
| Perfluorododecanesulfonic acid | PFDoS | 2018–19 | 79780-39-5 | 12 |
| **Fluorotelomer sulfonic acids** | | | | |
| 1*H*,1*H*, 2*H*, 2*H*-Perfluorohexane sulfonic acid | 4:2FTS | 2018–19 | 757124-72-4 | 6 |
| 1*H*,1*H*, 2*H*, 2*H*-Perfluorooctane sulfonic acid | 6:2FTS | 2018–19 | 27619-97-2 | 8 |
| 1*H*,1*H*, 2*H*, 2*H*-Perfluorodecane sulfonic acid | 8:2FTS | 2018–19 | 39108-34-4 | 10 |
| **Perfluorooctane sulfonamides** | | | | |
| Perfluorooctanesulfonamide | PFOSA | 2013–14 and 2018–19 | 754-91-6 | 8 |
| N-methyl perfluorooctanesulfonamide | N-MeFOSA | 2018–19 | 31506-32-8 | 8 |
| N-ethyl perfluorooctanesulfonamide | N-EtFOSA | 2018–19 | 4151-50-2 | 8 |
| **Perfluorooctane sulfonamidoacetic acids** | | | | |
| N-methyl perfluorooctanesulfonamidoacetic acid | N-MeFOSAA | 2018–19 | 2355-31-9 | 8 |
| N-ethyl perfluorooctanesulfonamidoacetic acid | N-EtFOSAA | 2018–19 | 2991-50-6 | 8 |
| **Perfluorooctane sulfonamide ethanols** | | | | |
| N-methyl perfluorooctanesulfonamidoethanol | N-MeFOSE | 2018–19 | 24448-09-7 | 8 |
| N-ethyl perfluorooctanesulfonamidoethanol | N-EtFOSE | 2018–19 | 1691-99-2 | 8 |
| **Per- and Polyfluoroether carboxylic acids** | | | | |
| Hexafluoropropylene oxide dimer acid | HFPO-DA | 2018–19 | 13252-13-6 | 3 |
| 4,8-Dioxa-3*H*-perfluorononanoic acid | ADONA | 2018–19 | 919005-14-4 | 3 |
| **Ether sulfonic acids** | | | | |
| 9-Chlorohexadecafluoro-3-oxanonane-1-sulfonic acid | 9Cl-PF3ONS | 2018–19 | 756426-58-1 | 6 |
| 11-Chloroeicosafluoro-3-oxaundecane-1-sulfonic acid | 11Cl-PF3OUdS | 2018–19 | 763051-92-9 | 8 |

^a^ CAS Number = Chemical Abstracts Service Registry Number

**Table A.4. Fish species collected for the 2013−2014 and 2018−2019 NRSA fish tissue studies.**

| **Common name** | **Scientific name^a^** | **2013−2014 NRSA** | | | **2018−2019 NRSA** | | |
| --- | --- | --- | --- | --- | --- | --- | --- |
|  |  | **Number of samples** | **Percent of samples** | **Length range (mm TL)^b^** | **Number of samples** | **Percent of samples** | **Length range (mm TL)** |
| Channel Catfish | *Ictalurus punctatus* | 84 | 23.8% | 258–718 | 62 | 21.4% | 230–736 |
| Smallmouth Bass | *Micropterus dolomieu* | 66 | 18.7% | 186–515 | 54 | 18.6% | 185–500 |
| Largemouth Bass | *Micropterus salmoides* | 37 | 10.5% | 206–485 | 42 | 14.5% | 213–545 |
| Walleye | *Sander vitreus* | 18 | 5.1% | 304–685 | 7 | 2.4% | 322–695 |
| Spotted Bass | *Micropterus punctulatus* | 15 | 4.2% | 186–410 | 10 | 3.4% | 188–392 |
| Blue Catfish | *Ictalurus furcatus* | 12 | 3.4% | 320–599 | 8 | 2.8% | 260–720 |
| Rainbow Trout | *Oncorhynchus mykiss* | 12 | 3.4% | 165–453 | 6 | 2.1% | 180–522 |
| Common Carp | *Cyprinus carpio* | 10 | 2.8% | 175–663 | 6 | 2.1% | 435–605 |
| Brown Trout | *Salmo trutta* | 9 | 2.5% | 275–630 | 5 | 1.7% | 273–542 |
| Flathead Catfish | *Pylodictis olivaris* | 9 | 2.5% | 300–882 | 13 | 4.5% | 205–630 |
| Northern Pike | *Esox lucius* | 8 | 2.3% | 255–830 | 5 | 1.7% | 350–737 |
| White Sucker | *Catostomus commersonii* | 8 | 2.3% | 217–460 | 10 | 3.4% | 194–530 |
| Mountain Whitefish | *Prosopium williamsoni* | 7 | 2.0% | 291–480 | 3 | 1.0% | 271–390 |
| Northern Pikeminnow | *Ptychocheilus oregonensis* | 5 | 1.4% | 245–470 | 1 | 0.3% | 222–282 |
| White Bass | *Morone chrysops* | 5 | 1.4% | 332–420 | 4 | 1.4% | 234–439 |
| Yellow Perch | *Perca flavescens* | 5 | 1.4% | 190–278 | 12 | 4.1% | 186–297 |
| Freshwater Drum | *Aplodinotus grunniens* | 4 | 1.1% | 248–642 | 7 | 2.4% | 295–472 |
| Largescale Sucker | *Catostomus macrocheilus* | 4 | 1.1% | 330–490 | 0 | 0.0% | NA |
| Rock Bass | *Ambloplites rupestris* | 4 | 1.1% | 175–221 | 8 | 2.8% | 186–237 |
| White Crappie | *Pomoxis annularis* | 4 | 1.1% | 205–355 | 3 | 1.0% | 249–327 |
| Smallmouth buffalo | *Ictiobus bubalus* | 3 | 0.8% | 332–425 | 0 | 0.0% | NA |
| Black Bullhead | *Ameiurus melas* | 2 | 0.6% | 205–273 | 0 | 0.0% | NA |
| Brook Trout | *Salvelinus fontinalis* | 2 | 0.6% | 210–280 | 1 | 0.3% | 241–317 |
| Brown Bullhead | *Ameiurus nebulosus* | 2 | 0.6% | 277–341 | 2 | 0.7% | 205–320 |
| Cutthroat Trout | *Oncorhynchus clarkii* | 2 | 0.6% | 309–380 | 1 | 0.3% | 225 |
| River Carpsucker | *Carpiodes carpio* | 2 | 0.6% | 291–395 | 0 | 0.0% | NA |
| Sauger | *Sander canadensis* | 2 | 0.6% | 308–487 | 1 | 0.3% | 400 |
| Striped Bass | *Morone saxatilis* | 2 | 0.6% | 380–760 | 1 | 0.3% | 374 |
| Striped Mullet | *Mugil cephalus* | 2 | 0.6% | 377–441 | 0 | 0.0% | NA |
| Bluegill | *Lepomis macrochirus* | 1 | 0.3% | 164–205 | 2 | 0.7% | 191–224 |
| Longnose Gar | *Lepisosteus osseus* | 1 | 0.3% | 620–750 | 0 | 0.0% | NA |
| Muskellunge | *Esox masquinongy* | 1 | 0.3% | 555 | 0 | 0.0% | NA |
| Redear Sunfish | *Lepomis microlophus* | 1 | 0.3% | 260–310 | 1 | 0.3% | 194 |
| Saugeye | *Sander vitreus x S. canadensis* | 1 | 0.3% | 393–479 | 0 | 0.0% | NA |
| White Catfish | *Ameiurus catus* | 1 | 0.3% | 285–300 | 0 | 0.0% | NA |
| White Perch | *Morone americana* | 1 | 0.3% | 183–205 | 1 | 0.3% | 251–264 |
| Yellow Bullhead | *Ameiurus natalis* | 1 | 0.3% | 174–228 | 0 | 0.0% | NA |
| Black Crappie | *Pomoxis nigromaculatus* | 0 | 0.0% | NA | 3 | 1.0% | 210–292 |
| Redbreast Sunfish | *Lepomis auritus* | 0 | 0.0% | NA | 2 | 0.7% | 190–252 |
| Silver Carp | *Hypophthalmichthys molitrix* | 0 | 0.0% | NA | 2 | 0.7% | 290–360 |
| Chain Pickerel | *Esox niger* | 0 | 0.0% | NA | 1 | 0.3% | 530–620 |
| Green Sunfish | *Lepomis cyanellus* | 0 | 0.0% | NA | 1 | 0.3% | 221 |
| Longear Sunfish | *Lepomis megalotis* | 0 | 0.0% | NA | 1 | 0.3% | 215–235 |
| Round Whitefish | *Prosopium cylindraceum* | 0 | 0.0% | NA | 1 | 0.3% | 285–350 |
| Shoal Bass | *Micropterus cataractae* | 0 | 0.0% | NA | 1 | 0.3% | 290–370 |
| Shortnose Gar | *Lepisosteus platostomus* | 0 | 0.0% | NA | 1 | 0.3% | 557–680 |
| Tyee Sucker | *Catostomus tsiltcoosensis* | 0 | 0.0% | NA | 1 | 0.3% | 305–390 |
| **Total** |  | **353** | 100% |  | **290** | 100% |  |

^a^ Scientific names follow: Page, L.M., Espinosa-Pérez, H., Findley, L.T., Gilbert, C.R., Lea, R.N., Mandrak, N.E., Mayden, R.L., Nelson, J.S., 2013. Common and Scientific Names of Fishes from the United States, Canada, and Mexico, 7th edition. American Fisheries Society Special Publication 34. Bethesda, MD. 243 pp. https://doi.org/10.47886/9781934874318.

^b^ mm TL = millimeters total length; NA = Not applicable

**Table A.5. QC data summary for the 2013–14 and 2018–19 NRSA mercury fish fillet sample results.**

| **QC operation^a^** | **Acceptance limit^b^** | **2013–14 exceptions (353 total results)** | **2018–19 exceptions (290 total results)** |
| --- | --- | --- | --- |
| Multi-point Calibration | RSD ≤ 15% | None | None |
| Calibration Verification | 70%–130% of nominal value | None | None |
| Bubbler Blank | ≤ 50 pg of mercury | None | None |
| Method Blank | ≤ 400 pg of mercury | None | None |
| Lab Control Sample | 70%–130% | None | None |
| MS/MSD Recovery | 70%– 130% | None | None |
| MS/MSD Precision | RPD ≤ 30% | None | None |
| QC Sample | 75%–125% | None | None |

^a^ MS/MSD = Matrix Spike/Matrix Spike Duplicate

^b^ RSD = Relative Standard Deviation; RPD = Relative Percent Difference; pg = picograms

**Table A.6. QC data summary for the 2013–14 and 2018–19 NRSA PCB fish fillet sample results.**

| **QC operation** | **Acceptance limit^a^** | **Issue^b^** | **2013–14 exceptions and implications (223 samples; 36,512 total sample/analyte results)** | **2018–19 exceptions and implications (290 samples; 46,400 total sample/analyte results)** |
| --- | --- | --- | --- | --- |
| Multi-point Calibration | RSD ≤ 20% | High RSD | 81 results were considered estimated values | None |
| Calibration Verification | 75%–125% for native congeners  50%–145% for labeled congeners | High verification results  Low verification results | 11 results were considered estimated concentrations  14 results may be false negatives  2 results were considered estimated concentrations | 12 results were considered estimated concentrations |
| Method Blank | ≤ 5x MDL | Congeners identified in the method blank above 5x the MDL | 215 results <5x the associated method blank result were considered non-detects  80 results >5x but <10x the associated method blank result were considered maximum values  447 results >10x the associated method blank results were not affected | 421 results <5x the associated method blank result were considered non-detects  160 results >5x but <10x the associated method blank result were considered maximum values  2216 results >10x the associated method blank results were not affected |
| Lab Control Sample | 60%–135% for native congeners  40%–145% for labeled congeners | LCS results outside of the limits | None | None |
| Labeled Compound Recovery | 5%–145% for mono- to tri-congeners and ^13^C_12_-2,3’,4’,5-tetra and   10%–145% for all other congeners | Labeled compounds with recoveries outside of the limits | 46 results with high labeled compound recoveries were considered estimated concentrations | 54 results with low labeled compound recoveries were not detected may be false negatives  100 results with low labeled compound recoveries were for analytes that were detected, and results were considered estimated concentrations |
| Ion Abundance Ratio | ± 15% of theoretical ratio for the congener | None | None | 346 results with high ion abundance ratios were considered estimated concentrations  195 results with low ion abundance ratios were considered estimated concentrations |
| Lab Duplicate Sample | ≤ 50% for concentrations greater than 5x MDL  < 100% for concentrations less than 5x MDL | High RPD | 6 results were for analytes that were detected and were considered estimated concentrations | None |

^a^ RSD = Relative Standard Deviation; MDL = Method Detection Limit

^b^ LCS = Lab Control Sample; RPD = Relative Percent Difference

**Table A.7.** **QC data summary for the 2013–14 NRSA PFAS fish fillet sample results.**

| **QC operation** | **Acceptance limit^a^** | **Issue^b^** | **2013–14 exceptions and implications (349 samples; 4,576 total sample/analyte results)** |
| --- | --- | --- | --- |
| Multi-point Calibration | RSD ≤ 35% | None | None |
| Calibration Verification | 50%–150% | High verification results for 2 analytes  Low verification results for 1 analyte | 20 results for analytes that were not detected were not affected  1 result for a detected analyte considered an estimated concentration  1 result for a detected analyte considered an estimated concentration |
| Method Blank | < 1/2 the quantitation limit for each analyte | Analytes identified in the method blank above 1/2 the quantitation limit | 123 results <5x the associated method blank result were considered non-detects  30 results >5x but <10x the associated method blank result were considered maximum values  229 results >10x the associated method blank results were not affected |
| Lab Control Sample | 60%–140% | Analytes with low LCS recovery | 1 analyte that was not detected may be a false negative |
| Labeled Compound Recovery | 25%–150% | Labeled compounds with recoveries outside of the limits | 9 results with high labeled compound recoveries were for analytes there were not detected, and results were not affected  11 results with high labeled compound recoveries were for analytes there were detected, and results were considered estimated concentrations  13 results with low labeled compound recoveries were not detected may be false negatives  2 results with low labeled compound recoveries were for analytes that were detected, and results were considered estimated concentrations |
| Lab Duplicate Sample | ≤ 30% | High RPD | 6 results were for analytes that were detected and were considered estimated concentrations  1 result was for an analyte that was converted to a non-detect because of method blank concerns |

^a^ RSD = Relative Standard Deviation

^b^ RPD = Relative Percent Difference

**Table A.8. QC data summary for the 2018–19 NRSA PFAS fish fillet sample results.**

| **QC operation** | **Acceptance limit** | **Issue^a^** | **2018–19 exceptions and implications (290 samples; 9,570 total sample/analyte results)** |
| --- | --- | --- | --- |
| Multi-point Calibration | r^2^ ≥ 0.95 | None | None |
| Calibration Verification | 70%–130% | None | None |
| Method Blank | ≤ MDL for each analyte | Analytes identified in the method blank above the MDL | 45 results <5x the associated method blank result were considered non-detects  5 results >5x but <10x the associated method blank result were considered maximum values  14 results >10x the associated method blank results were not affected |
| Lab Control Sample | 40%–160% overall, but varied by analyte | Analytes with high LCS recovery  Analytes with low LCS recovery | 16 results that may reflect a high bias  182 results that were not detected were not affected  128 results that were not detected may be false negatives  6 results that were detected were considered estimated concentrations |
| Labeled Compound Recovery | 50%–150% | Labeled compounds with recoveries outside of the limits | 178 results with high labeled compound recoveries were for analytes there were not detected, and results were not affected  3 results with high labeled compound recoveries were for analytes there were detected, and results were considered estimated concentrations  87 results with low labeled compound recoveries were not detected may be false negatives  112 results with low labeled compound recoveries were for analytes that were detected, and results were considered estimated concentrations |
| Lab Duplicate Sample | ≤ 40% | High RPD | 26 results were not affected  10 results were for analytes that were detected and were considered estimated concentrations |

^a^ MDL = Method Detection Limit; LCS = Lab Control Sample; RPD = Relative Percent Difference

| **Table A.9. Total mercury results (ng/g, wet weight) for 2013–14 NRSA fish fillet tissue samples.** | | | | | | | | | |
| --- | --- | --- | --- | --- | --- | --- | --- | --- | --- |
| **Chemical** | **Number of detections** | **MDL^a^** | **Percentile^b^** | | | | **Maximum** | **Frequency of occurrence^c^** |  |
|  |  |  | **25^th^** | **50^th^** | **75^th^** | **90^th^** |  |  |  |
| Total mercury | 353 | 0.06 | 111 | 180 | 288 | 461 | 1070 | 100.0% |  |
| ^a^ MDL= Method Detection Limit in ng/g, wet weight  ^b^ Percentile concentrations are statistical estimates for the sampled population of river length determined for the study (a length of 79,448 km); maximum concentration is the maximum value measured at 353 sites.  ^c^ Percent frequency of occurrence values are based on 353 possible detections. | | | | | | | | | |

| **Table A.10. Total mercury results (ng/g, wet weight) for 2018–19 NRSA fish fillet tissue samples.** | | | | | | | | | |
| --- | --- | --- | --- | --- | --- | --- | --- | --- | --- |
| **Chemical** | **Number of detections** | **MDL^a^** | **Percentile^b^** | | | | **Maximum** | **Frequency of occurrence^c^** |  |
|  |  |  | **25^th^** | **50^th^** | **75^th^** | **90^th^** |  |  |  |
| Total mercury | 290 | 0.09 | 101 | 180 | 315 | 495 | 1340 | 100.0% |  |
| ^a^ MDL= Method Detection Limit in ng/g, wet weight  ^b^ Percentile concentrations are statistical estimates for the sampled population of river length determined for the study (a length of 66,142 km); maximum concentration is the maximum value measured at 290 sites.  ^c^ Percent frequency of occurrence values are based on 290 possible detections. | | | | | | | | | |

| **Table A.11. PCB results (ng/g, wet weight) for 2013–14 NRSA fish fillet tissue samples.** | | | | | | | | |  |
| --- | --- | --- | --- | --- | --- | --- | --- | --- | --- |
| **Chemical** | **Number of detections** | **MDL^a^** | **Percentile^b^** | | | | **Maximum** | **Frequency of occurrence^c^** | |
|  |  |  | **25^th^** | **50^th^** | **75^th^** | **90^th^** |  |  |  |
| PCB 1 | 103 | 0.00006 | < MDL | < MDL | 0.000580 | 0.00223 | 114 | 46.2% | |
| PCB 2 | 65 | 0.00009 | < MDL | < MDL | 0.000118 | 0.000438 | 0.169 | 29.1% | |
| PCB 3 | 85 | 0.00010 | < MDL | < MDL | 0.000284 | 0.000739 | 2.38 | 38.1% | |
| PCB 4 | 142 | 0.00010 | < MDL | 0.00124 | 0.00984 | 0.0706 | 317 | 63.7% | |
| PCB 5 | 54 | 0.00007 | < MDL | < MDL | < MDL | 0.000535 | 0.0278 | 24.2% | |
| PCB 6 | 156 | 0.00011 | < MDL | 0.000641 | 0.00427 | 0.0173 | 18.2 | 70.0% | |
| PCB 7 | 92 | 0.00009 | < MDL | < MDL | 0.000451 | 0.00237 | 0.423 | 41.3% | |
| PCB 8 | 188 | 0.00010 | 0.000713 | 0.00344 | 0.0151 | 0.0556 | 59.3 | 84.3% | |
| PCB 9 | 109 | 0.00010 | < MDL | < MDL | 0.000924 | 0.00316 | 4.44 | 48.9% | |
| PCB 10 | 71 | 0.00008 | < MDL | < MDL | 0.000421 | 0.00317 | 55.3 | 31.8% | |
| PCB 11 | 92 | 0.00033 | < MDL | < MDL | 0.00434 | 0.0126 | 0.733 | 41.3% | |
| PCB 12 + 13 | 62 | 0.00020 | < MDL | < MDL | 0.000269 | 0.00143 | 1.23 | 27.8% | |
| PCB 14 | 8 | 0.00009 | < MDL | < MDL | < MDL | < MDL | 0.00766 | 3.6% | |
| PCB 15 | 163 | 0.00013 | < MDL | 0.000950 | 0.00399 | 0.0101 | 0.811 | 73.1% | |
| PCB 16 | 210 | 0.00017 | 0.000933 | 0.00370 | 0.0182 | 0.121 | 2.55 | 94.2% | |
| PCB 17 | 211 | 0.00007 | 0.00133 | 0.00677 | 0.0478 | 0.267 | 40.3 | 94.6% | |
| PCB 18 + 30 | 209 | 0.00017 | 0.00277 | 0.0129 | 0.0799 | 0.283 | 36.0 | 93.7% | |
| PCB 19 | 183 | 0.00015 | 0.000267 | 0.00110 | 0.00728 | 0.0558 | 119 | 82.1% | |
| PCB 20 + 28 | 221 | 0.00023 | 0.0189 | 0.0770 | 0.533 | 1.41 | 51.8 | 99.1% | |
| PCB 21 + 33 | 210 | 0.00020 | 0.00144 | 0.00537 | 0.0177 | 0.0515 | 3.43 | 94.2% | |
| PCB 22 | 218 | 0.00011 | 0.00339 | 0.0124 | 0.0860 | 0.212 | 11.1 | 97.8% | |
| PCB 23 | 76 | 0.00010 | < MDL | < MDL | 0.000189 | 0.000628 | 0.194 | 34.1% | |
| PCB 24 | 129 | 0.00013 | < MDL | 0.000203 | 0.00153 | 0.00632 | 1.41 | 57.8% | |
| PCB 25 | 217 | 0.00009 | 0.000601 | 0.00246 | 0.0218 | 0.0575 | 8.97 | 97.3% | |
| PCB 26 + 29 | 215 | 0.00020 | 0.00132 | 0.00633 | 0.0418 | 0.136 | 32.3 | 96.4% | |
| PCB 27 | 196 | 0.00009 | 0.000246 | 0.00112 | 0.00979 | 0.0519 | 65.2 | 87.9% | |
| PCB 31 | 222 | 0.00014 | 0.00908 | 0.0370 | 0.182 | 0.515 | 51.9 | 99.6% | |
| PCB 32 | 208 | 0.00008 | 0.000823 | 0.00417 | 0.0322 | 0.129 | 47.9 | 93.3% | |
| PCB 34 | 129 | 0.00012 | < MDL | 0.000264 | 0.00158 | 0.00785 | 3.66 | 57.8% | |
| PCB 35 | 11 | 0.00016 | < MDL | < MDL | < MDL | < MDL | 0.00590 | 4.9% | |
| PCB 36 | 32 | 0.00011 | < MDL | < MDL | < MDL | 0.000563 | 0.156 | 14.3% | |
| PCB 37 | 198 | 0.00017 | 0.000659 | 0.00277 | 0.0127 | 0.0284 | 4.74 | 88.8% | |
| PCB 38 | 109 | 0.00013 | < MDL | < MDL | 0.000751 | 0.00245 | 0.184 | 48.9% | |
| PCB 39 | 122 | 0.00017 | < MDL | 0.000225 | 0.00177 | 0.00431 | 0.177 | 54.7% | |
| PCB 40 + 41 + 71 | 217 | 0.00043 | 0.00470 | 0.0218 | 0.152 | 0.607 | 30.2 | 97.3% | |
| PCB 42 | 222 | 0.00016 | 0.00528 | 0.0317 | 0.172 | 0.744 | 29.1 | 99.6% | |
| PCB 43 | 187 | 0.00015 | 0.000351 | 0.00172 | 0.0165 | 0.0644 | 5.92 | 83.9% | |
| PCB 44 + 47 + 65 | 223 | 0.00034 | 0.0294 | 0.147 | 0.882 | 2.82 | 261 | 100.0% | |
| PCB 45 + 51 | 205 | 0.00026 | 0.00138 | 0.00702 | 0.0453 | 0.249 | 15.0 | 91.9% | |
| PCB 46 | 160 | 0.00015 | < MDL | 0.000933 | 0.00761 | 0.0452 | 1.23 | 71.7% | |
| PCB 48 | 216 | 0.00012 | 0.00183 | 0.00936 | 0.0588 | 0.227 | 4.62 | 96.9% | |
| PCB 49 + 69 | 223 | 0.00028 | 0.0220 | 0.109 | 0.734 | 2.41 | 183 | 100.0% | |
| PCB 50 + 53 | 203 | 0.00018 | 0.000692 | 0.00450 | 0.0373 | 0.144 | 21.1 | 91.0% | |
| PCB 52 | 223 | 0.00011 | 0.0360 | 0.202 | 1.23 | 3.62 | 284 | 100.0% | |
| PCB 54 | 86 | 0.00010 | < MDL | < MDL | 0.000590 | 0.00337 | 1.21 | 38.6% | |
| PCB 55 | 132 | 0.00012 | < MDL | 0.000287 | 0.00146 | 0.00570 | 4.00 | 59.2% | |
| PCB 56 | 223 | 0.00013 | 0.00552 | 0.0252 | 0.104 | 0.344 | 83.7 | 100.0% | |
| PCB 57 | 171 | 0.00014 | < MDL | 0.000609 | 0.00361 | 0.00925 | 4.67 | 76.7% | |
| PCB 58 | 150 | 0.00011 | < MDL | 0.000298 | 0.00231 | 0.0146 | 1.41 | 67.3% | |
| PCB 59 + 62 + 75 | 217 | 0.00034 | 0.00249 | 0.0120 | 0.0710 | 0.264 | 16.9 | 97.3% | |
| PCB 60 | 222 | 0.00011 | 0.00748 | 0.0329 | 0.204 | 0.483 | 93.4 | 99.6% | |
| PCB 61 + 70 + 74 + 76 | 222 | 0.00043 | 0.0594 | 0.221 | 1.11 | 2.93 | 408 | 99.6% | |
| PCB 63 | 220 | 0.00010 | 0.00212 | 0.00887 | 0.0596 | 0.178 | 29.6 | 98.7% | |
| PCB 64 | 223 | 0.00016 | 0.0137 | 0.0702 | 0.426 | 1.41 | 84.7 | 100.0% | |
| PCB 66 | 222 | 0.00017 | 0.0478 | 0.179 | 1.15 | 3.48 | 316 | 99.6% | |
| PCB 67 | 198 | 0.00011 | 0.000426 | 0.00179 | 0.0119 | 0.0302 | 9.78 | 88.8% | |
| PCB 68 | 211 | 0.00018 | 0.000740 | 0.00292 | 0.00134 | 0.0632 | 5.99 | 94.6% | |
| PCB 72 | 208 | 0.00010 | 0.000632 | 0.00259 | 0.0137 | 0.0591 | 10.0 | 93.3% | |
| PCB 73 | 57 | 0.00011 | < MDL | < MDL | 0.000171 | 0.00432 | 4.80 | 25.6% | |
| PCB 77 | 211 | 0.00026 | 0.00125 | 0.00416 | 0.0153 | 0.0438 | 15.5 | 94.6% | |
| PCB 78 | 3 | 0.00019 | < MDL | < MDL | < MDL | < MDL | 0.00414 | 1.4% | |
| PCB 79 | 166 | 0.00015 | < MDL | 0.00126 | 0.00673 | 0.0325 | 0.635 | 74.4% | |
| PCB 80 | 18 | 0.00012 | < MDL | < MDL | < MDL | < MDL | 0.0171 | 8.1% | |
| PCB 81 | 143 | 0.00020 | < MDL | 0.000505 | 0.00285 | 0.00866 | 1.16 | 64.1% | |
| PCB 82 | 215 | 0.00016 | 0.00349 | 0.0201 | 0.0634 | 0.364 | 24.8 | 96.4% | |
| PCB 83 + 99 | 223 | 0.00021 | 0.111 | 0.445 | 2.16 | 6.98 | 205 | 100.0% | |
| PCB 84 | 218 | 0.00016 | 0.00496 | 0.0226 | 0.118 | 0.549 | 10.4 | 97.8% | |
| PCB 85 + 116 + 117 | 222 | 0.00035 | 0.140 | 0.593 | 2.30 | 9.99 | 274 | 99.6% | |
| PCB 86 + 87 + 97 + 109 + 119 + 125 | 221 | 0.00059 | 0.0461 | 0.229 | 0.889 | 3.58 | 142 | 99.1% | |
| PCB 88 + 91 | 221 | 0.00024 | 0.0105 | 0.0449 | 0.271 | 0.983 | 32.0 | 99.1% | |
| PCB 89 | 146 | 0.00010 | < MDL | 0.000546 | 0.00328 | 0.0139 | 0.945 | 65.5% | |
| PCB 90 + 101 + 113 | 223 | 0.00030 | 0.123 | 0.474 | 1.67 | 8.03 | 229 | 100.0% | |
| PCB 92 | 223 | 0.00011 | 0.0229 | 0.0853 | 0.404 | 1.62 | 53.7 | 100.0% | |
| PCB 93 + 98 + 100 + 102 | 198 | 0.00044 | 0.00183 | 0.00865 | 0.0532 | 0.194 | 12.1 | 88.8% | |
| PCB 94 | 141 | 0.00014 | < MDL | 0.000487 | 0.00596 | 0.0263 | 3.51 | 63.2% | |
| PCB 95 | 222 | 0.00012 | 0.0345 | 0.147 | 0.576 | 2.82 | 82.3 | 99.6% | |
| PCB 96 | 150 | 0.00012 | < MDL | 0.000295 | 0.00162 | 0.00744 | 0.524 | 67.3% | |
| PCB 103 | 193 | 0.00011 | 0.000619 | 0.00241 | 0.0168 | 0.0814 | 3.92 | 86.5% | |
| PCB 104 | 66 | 0.00010 | < MDL | < MDL | 0.000139 | 0.000840 | 0.0837 | 29.6% | |
| PCB 105 | 223 | 0.00019 | 0.0530 | 0.217 | 0.818 | 2.72 | 123 | 100.0% | |
| PCB 106 | 5 | 0.00032 | < MDL | < MDL | < MDL | < MDL | 0.00355 | 2.2% | |
| PCB 107 | 221 | 0.00015 | 0.0118 | 0.0450 | 0.203 | 0.673 | 27.6 | 99.1% | |
| PCB 108 + 124 | 220 | 0.00023 | 0.00385 | 0.0125 | 0.0433 | 0.132 | 13.7 | 98.7% | |
| PCB 110 + 115 | 202 | 0.00025 | 0.00234 | 0.00959 | 0.0552 | 0.204 | 13.1 | 90.6% | |
| PCB 111 | 160 | 0.00016 | < MDL | 0.000557 | 0.00278 | 0.0111 | 0.900 | 71.7% | |
| PCB 112 | 2 | 0.00009 | < MDL | < MDL | < MDL | < MDL | 1.94 | 0.9% | |
| PCB 114 | 216 | 0.00016 | 0.00297 | 0.0129 | 0.0528 | 0.151 | 12.4 | 96.9% | |
| PCB 118 | 223 | 0.00019 | 0.161 | 0.650 | 2.89 | 8.43 | 259 | 100.0% | |
| PCB 120 | 205 | 0.00012 | 0.000966 | 0.00365 | 0.0140 | 0.0618 | 1.12 | 91.9% | |
| PCB 121 | 150 | 0.00013 | < MDL | 0.000417 | 0.00178 | 0.00577 | 0.496 | 67.3% | |
| PCB 122 | 187 | 0.00012 | 0.000547 | 0.00321 | 0.0121 | 0.0414 | 3.81 | 83.9% | |
| PCB 123 | 217 | 0.00014 | 0.00272 | 0.0110 | 0.0570 | 0.133 | 8.95 | 97.3% | |
| PCB 126 | 188 | 0.00030 | 0.000473 | 0.00146 | 0.00542 | 0.0150 | 0.341 | 84.3% | |
| PCB 127 | 33 | 0.00022 | < MDL | < MDL | < MDL | 0.00157 | 0.0366 | 14.8% | |
| PCB 128 + 166 | 223 | 0.00013 | 0.0453 | 0.182 | 0.603 | 1.87 | 20.0 | 100.0% | |
| PCB 129 + 138 + 160 + 163 | 223 | 0.00098 | 0.374 | 1.34 | 4.41 | 16.1 | 185 | 100.0% | |
| PCB 130 | 222 | 0.00012 | 0.0136 | 0.0606 | 0.204 | 0.777 | 13.3 | 99.6% | |
| PCB 131 | 184 | 0.00011 | 0.000460 | 0.00419 | 0.0129 | 0.0616 | 1.46 | 82.5% | |
| PCB 132 | 222 | 0.00008 | 0.0204 | 0.104 | 0.344 | 1.71 | 24.6 | 99.6% | |
| PCB 133 | 216 | 0.00007 | 0.00476 | 0.0180 | 0.0777 | 0.359 | 7.44 | 96.9% | |
| PCB 134 + 143 | 184 | 0.00019 | 0.000851 | 0.0103 | 0.0382 | 0.217 | 5.79 | 82.5% | |
| PCB 135 + 151 | 222 | 0.00022 | 0.0351 | 0.162 | 0.702 | 3.27 | 41.5 | 99.6% | |
| PCB 136 | 218 | 0.00014 | 0.00354 | 0.0184 | 0.0626 | 0.259 | 5.95 | 97.8% | |
| PCB 137 | 220 | 0.00023 | 0.0117 | 0.0466 | 0.176 | 0.590 | 13.0 | 98.7% | |
| PCB 139 + 140 | 193 | 0.00015 | 0.00259 | 0.0110 | 0.0437 | 0.216 | 3.99 | 86.5% | |
| PCB 141 | 223 | 0.00013 | 0.0297 | 0.136 | 0.457 | 1.86 | 24.9 | 100.0% | |
| PCB 142 | 14 | 0.00020 | < MDL | < MDL | < MDL | < MDL | 0.0867 | 6.3% | |
| PCB 144 | 220 | 0.00010 | 0.00464 | 0.0190 | 0.0708 | 0.282 | 25.7 | 98.7% | |
| PCB 145 | 58 | 0.00012 | < MDL | < MDL | < MDL | 0.000429 | 0.0153 | 26.0% | |
| PCB 146 | 222 | 0.00006 | 0.0575 | 0.240 | 0.752 | 3.30 | 35.2 | 99.6% | |
| PCB 147 + 149 | 223 | 0.00021 | 0.102 | 0.525 | 1.90 | 8.18 | 106 | 100.0% | |
| PCB 148 | 191 | 0.00010 | 0.000385 | 0.00149 | 0.00900 | 0.0324 | 0.701 | 85.7% | |
| PCB 150 | 175 | 0.00012 | 0.000127 | 0.000496 | 0.00287 | 0.0128 | 0.229 | 78.5% | |
| PCB 152 | 130 | 0.00010 | < MDL | 0.000134 | 0.000772 | 0.00291 | 0.189 | 58.3% | |
| PCB 153 + 168 | 223 | 0.00018 | 0.393 | 1.39 | 6.45 | 19.8 | 181 | 100.0% | |
| PCB 154 | 219 | 0.00012 | 0.00361 | 0.0131 | 0.0681 | 0.264 | 3.83 | 98.2% | |
| PCB 155 | 192 | 0.00013 | 0.000243 | 0.000834 | 0.00403 | 0.0114 | 0.489 | 86.1% | |
| PCB 156 + 157 | 223 | 0.00041 | 0.0306 | 0.118 | 0.380 | 1.11 | 25.9 | 100.0% | |
| PCB 158 | 221 | 0.00009 | 0.0221 | 0.103 | 0.296 | 1.12 | 11.8 | 99.1% | |
| PCB 159 | 212 | 0.00013 | 0.00111 | 0.00506 | 0.0205 | 0.0856 | 0.800 | 95.1% | |
| PCB 161 | 8 | 0.00011 | < MDL | < MDL | < MDL | < MDL | 0.00298 | 3.6% | |
| PCB 162 | 215 | 0.00016 | 0.00121 | 0.00406 | 0.0150 | 0.0563 | 0.859 | 96.4% | |
| PCB 164 | 222 | 0.00013 | 0.0112 | 0.0489 | 0.168 | 0.685 | 9.44 | 99.6% | |
| PCB 165 | 158 | 0.00009 | < MDL | 0.000496 | 0.00296 | 0.0109 | 1.95 | 70.9% | |
| PCB 167 | 223 | 0.00016 | 0.0136 | 0.0457 | 0.185 | 0.598 | 9.23 | 100.0% | |
| PCB 169 | 206 | 0.00025 | 0.000619 | 0.00211 | 0.00635 | 0.0196 | 0.194 | 92.4% | |
| PCB 170 | 222 | 0.00017 | 0.0616 | 0.221 | 0.824 | 3.25 | 44.3 | 99.6% | |
| PCB 171 + 173 | 221 | 0.00023 | 0.0147 | 0.0603 | 0.261 | 0.976 | 12.5 | 99.1% | |
| PCB 172 | 221 | 0.00010 | 0.0121 | 0.0432 | 0.169 | 0.717 | 9.21 | 99.1% | |
| PCB 174 | 223 | 0.00017 | 0.0364 | 0.142 | 0.621 | 2.52 | 30.8 | 100.0% | |
| PCB 175 | 207 | 0.00016 | 0.00226 | 0.00770 | 0.0302 | 0.127 | 1.40 | 92.8% | |
| PCB 176 | 211 | 0.00012 | 0.00172 | 0.00921 | 0.0415 | 0.152 | 1.62 | 94.6% | |
| PCB 177 | 223 | 0.00010 | 0.0250 | 0.122 | 0.455 | 1.42 | 22.8 | 100.0% | |
| PCB 178 | 223 | 0.00014 | 0.0135 | 0.0533 | 0.224 | 0.769 | 13.1 | 100.0% | |
| PCB 179 | 223 | 0.00014 | 0.00867 | 0.0396 | 0.169 | 0.670 | 7.00 | 100.0% | |
| PCB 180 + 193 | 222 | 0.00021 | 0.176 | 0.687 | 2.31 | 9.67 | 125 | 99.6% | |
| PCB 181 | 200 | 0.00010 | 0.000531 | 0.00228 | 0.00853 | 0.0292 | 0.918 | 89.7% | |
| PCB 182 | 193 | 0.00013 | 0.000487 | 0.00167 | 0.00638 | 0.0200 | 4.97 | 86.5% | |
| PCB 183 + 185 | 222 | 0.00019 | 0.0460 | 0.171 | 0.736 | 2.82 | 35.2 | 99.6% | |
| PCB 184 | 189 | 0.00015 | 0.000352 | 0.00145 | 0.00510 | 0.0113 | 0.556 | 84.8% | |
| PCB 186 | 14 | 0.00012 | < MDL | < MDL | < MDL | < MDL | 0.00407 | 6.3% | |
| PCB 187 | 223 | 0.00017 | 0.136 | 0.490 | 1.82 | 7.21 | 97.5 | 100.0% | |
| PCB 188 | 169 | 0.00008 | < MDL | 0.000666 | 0.00333 | 0.00894 | 0.179 | 75.8% | |
| PCB 189 | 215 | 0.00019 | 0.00233 | 0.00833 | 0.0302 | 0.108 | 1.47 | 96.4% | |
| PCB 190 | 223 | 0.00011 | 0.0132 | 0.0503 | 0.196 | 0.604 | 8.97 | 100.0% | |
| PCB 191 | 219 | 0.00012 | 0.00209 | 0.00721 | 0.0310 | 0.124 | 1.76 | 98.2% | |
| PCB 192 | 30 | 0.00017 | < MDL | < MDL | < MDL | 0.000350 | 0.00717 | 13.5% | |
| PCB 194 | 223 | 0.00012 | 0.0337 | 0.120 | 0.443 | 1.68 | 20.1 | 100.0% | |
| PCB 195 | 223 | 0.00015 | 0.0106 | 0.0409 | 0.161 | 0.555 | 8.06 | 100.0% | |
| PCB 196 | 221 | 0.00014 | 0.0140 | 0.0505 | 0.215 | 0.805 | 7.29 | 99.1% | |
| PCB 197 | 206 | 0.00018 | 0.00122 | 0.00406 | 0.0166 | 0.0662 | 0.532 | 92.4% | |
| PCB 198 + 199 | 223 | 0.00023 | 0.0455 | 0.162 | 0.554 | 2.24 | 22.5 | 100.0% | |
| PCB 200 | 205 | 0.00029 | 0.00137 | 0.00576 | 0.0246 | 0.0968 | 1.25 | 91.9% | |
| PCB 201 | 217 | 0.00010 | 0.00411 | 0.0147 | 0.0611 | 0.231 | 2.28 | 97.3% | |
| PCB 202 | 223 | 0.00014 | 0.00936 | 0.0343 | 0.153 | 0.502 | 7.57 | 100.0% | |
| PCB 203 | 222 | 0.00012 | 0.0326 | 0.125 | 0.486 | 1.54 | 13.3 | 99.6% | |
| PCB 204 | 129 | 0.00010 | < MDL | 0.000158 | 0.000774 | 0.00222 | 0.0494 | 57.8% | |
| PCB 205 | 215 | 0.00014 | 0.00159 | 0.00561 | 0.0208 | 0.0705 | 0.836 | 96.4% | |
| PCB 206 | 222 | 0.00013 | 0.0226 | 0.0854 | 0.319 | 0.782 | 12.5 | 99.6% | |
| PCB 207 | 217 | 0.00008 | 0.00262 | 0.00788 | 0.0328 | 0.0930 | 1.17 | 97.3% | |
| PCB 208 | 221 | 0.00009 | 0.00686 | 0.0273 | 0.103 | 0.297 | 6.00 | 99.1% | |
| PCB 209 | 221 | 0.00013 | 0.00997 | 0.0429 | 0.199 | 0.483 | 5.77 | 99.1% | |
| **Total PCBs** | **223** |  | **3.58** | **12.3** | **57.7** | **171** | **4617** | **100.0%** | |
| ^a^ MDL= Method Detection Limit in ng/g, wet weight  ^b^ Percentile concentrations are statistical estimates for the sampled population of river length determined for the study (a length of 48,826 km); maximum concentrations are the maximum values measured at 223 sites.  ^c^ Percent frequency of occurrence values are based on 223 possible detections. | | | | | | | | |  |

| **Table A.12. PCB results (ng/g, wet weight) for 2018–19 NRSA fish fillet tissue samples.** | | | | | | | | | |
| --- | --- | --- | --- | --- | --- | --- | --- | --- | --- |
| **Chemical** | **Number of detections** | **MDL^a^** | **Percentile^b^** | | | | **Maximum** | **Frequency of occurrence^c^** |  |
|  |  |  | **25^th^** | **50^th^** | **75^th^** | **90^th^** |  |  |  |
| PCB 1 | 49 | 0.00124 | < MDL | < MDL | < MDL | 0.00178 | 0.0797 | 16.9% |  |
| PCB 2 | 19 | 0.00063 | < MDL | < MDL | < MDL | < MDL | 0.00303 | 6.6% |  |
| PCB 3 | 40 | 0.00124 | < MDL | < MDL | < MDL | 0.00154 | 0.0195 | 13.8% |  |
| PCB 4 | 167 | 0.00107 | < MDL | 0.00149 | 0.00803 | 0.0237 | 2.05 | 57.6% |  |
| PCB 5 | 14 | 0.00068 | < MDL | < MDL | < MDL | < MDL | 0.00891 | 4.8% |  |
| PCB 6 | 134 | 0.00089 | < MDL | < MDL | 0.00307 | 0.00856 | 0.206 | 46.2% |  |
| PCB 7 | 29 | 0.00188 | < MDL | < MDL | < MDL | < MDL | 0.027 | 10.0% |  |
| PCB 8 | 209 | 0.00094 | < MDL | 0.00224 | 0.0104 | 0.0236 | 0.436 | 72.1% |  |
| PCB 9 | 50 | 0.00102 | < MDL | < MDL | < MDL | 0.00172 | 0.0306 | 17.2% |  |
| PCB 10 | 54 | 0.00096 | < MDL | < MDL | < MDL | 0.00162 | 0.103 | 18.6% |  |
| PCB 11 | 68 | 0.00561 | < MDL | < MDL | < MDL | 0.0127 | 0.896 | 23.4% |  |
| PCB 12 + 13 | 48 | 0.00086 | < MDL | < MDL | < MDL | 0.00139 | 0.0184 | 16.6% |  |
| PCB 14 | 4 | 0.00042 | < MDL | < MDL | < MDL | < MDL | 0.00465 | 1.4% |  |
| PCB 15 | 189 | 0.00086 | < MDL | 0.00131 | 0.00458 | 0.00987 | 0.277 | 65.2% |  |
| PCB 16 | 183 | 0.00172 | < MDL | 0.00313 | 0.0148 | 0.0343 | 1.08 | 63.1% |  |
| PCB 17 | 233 | 0.00118 | 0.00162 | 0.00465 | 0.0319 | 0.0729 | 1.90 | 80.3% |  |
| PCB 18 + 30 | 275 | 0.00118 | 0.00299 | 0.00898 | 0.0435 | 0.115 | 4.82 | 94.8% |  |
| PCB 19 | 147 | 0.00131 | < MDL | < MDL | 0.00752 | 0.0278 | 2.30 | 50.7% |  |
| PCB 20 + 28 | 285 | 0.00210 | 0.0135 | 0.0469 | 0.243 | 0.592 | 13.4 | 98.3% |  |
| PCB 21 + 33 | 203 | 0.00069 | < MDL | 0.00470 | 0.0160 | 0.0333 | 1.49 | 70.0% |  |
| PCB 22 | 273 | 0.00086 | 0.00281 | 0.0103 | 0.0472 | 0.0936 | 1.71 | 94.1% |  |
| PCB 23 | 4 | 0.00067 | < MDL | < MDL | < MDL | < MDL | 0.00163 | 1.4% |  |
| PCB 24 | 45 | 0.00178 | < MDL | < MDL | < MDL | 0.00314 | 0.0593 | 15.5% |  |
| PCB 25 | 207 | 0.00069 | < MDL | 0.00192 | 0.00892 | 0.0286 | 0.411 | 71.4% |  |
| PCB 26 + 29 | 252 | 0.00069 | 0.00141 | 0.00465 | 0.0208 | 0.0540 | 0.961 | 86.9% |  |
| PCB 27 | 140 | 0.00121 | < MDL | < MDL | 0.00757 | 0.0187 | 0.492 | 48.3% |  |
| PCB 31 | 252 | 0.00081 | 0.00657 | 0.0233 | 0.104 | 0.208 | 3.86 | 86.9% |  |
| PCB 32 | 210 | 0.00099 | < MDL | 0.00292 | 0.0149 | 0.0531 | 1.71 | 72.4% |  |
| PCB 34 | 109 | 0.00061 | < MDL | < MDL | 0.00124 | 0.00329 | 0.0907 | 37.6% |  |
| PCB 35 | 0 | 0.00060 | < MDL | < MDL | < MDL | < MDL | 0.00 | 0.0% |  |
| PCB 36 | 1 | 0.00072 | < MDL | < MDL | < MDL | < MDL | 0.000967 | 0.3% |  |
| PCB 37 | 239 | 0.00084 | 0.00122 | 0.00289 | 0.0103 | 0.0220 | 0.252 | 82.4% |  |
| PCB 38 | 66 | 0.00054 | < MDL | < MDL | < MDL | 0.00162 | 0.0452 | 22.8% |  |
| PCB 39 | 143 | 0.00022 | < MDL | < MDL | 0.000827 | 0.00298 | 0.112 | 49.3% |  |
| PCB 40 + 41 + 71 | 258 | 0.00183 | 0.00476 | 0.0146 | 0.0690 | 0.191 | 6.40 | 89.0% |  |
| PCB 42 | 260 | 0.00183 | 0.00413 | 0.0141 | 0.0803 | 0.222 | 15.8 | 89.7% |  |
| PCB 43 | 167 | 0.00141 | < MDL | 0.00180 | 0.00801 | 0.0265 | 1.19 | 57.6% |  |
| PCB 44 + 47 + 65 | 234 | 0.00183 | 0.0170 | 0.0720 | 0.386 | 1.01 | 54.4 | 80.7% |  |
| PCB 45 + 51 | 210 | 0.00138 | < MDL | 0.00437 | 0.0265 | 0.0771 | 3.49 | 72.4% |  |
| PCB 46 | 129 | 0.00138 | < MDL | < MDL | 0.00441 | 0.0155 | 0.500 | 44.5% |  |
| PCB 48 | 236 | 0.00138 | 0.00204 | 0.00680 | 0.0294 | 0.0765 | 3.36 | 81.4% |  |
| PCB 49 + 69 | 289 | 0.00141 | 0.0156 | 0.0512 | 0.313 | 0.770 | 49.0 | 99.7% |  |
| PCB 50 + 53 | 205 | 0.00138 | < MDL | 0.00315 | 0.0188 | 0.0592 | 1.82 | 70.7% |  |
| PCB 52 | 287 | 0.00150 | 0.0276 | 0.0849 | 0.434 | 0.979 | 58.3 | 99.0% |  |
| PCB 54 | 74 | 0.00090 | < MDL | < MDL | < MDL | 0.00301 | 0.421 | 25.5% |  |
| PCB 55 | 94 | 0.00111 | < MDL | < MDL | 0.00214 | 0.00943 | 0.146 | 32.4% |  |
| PCB 56 | 284 | 0.00049 | 0.00547 | 0.0180 | 0.0570 | 0.162 | 3.28 | 97.9% |  |
| PCB 57 | 107 | 0.00069 | < MDL | < MDL | 0.00159 | 0.00423 | 0.0394 | 36.9% |  |
| PCB 58 | 116 | 0.00093 | < MDL | < MDL | 0.00199 | 0.00566 | 0.262 | 40.0% |  |
| PCB 59 + 62 + 75 | 219 | 0.00183 | 0.00205 | 0.00655 | 0.0327 | 0.0827 | 4.00 | 75.5% |  |
| PCB 60 | 289 | 0.00049 | 0.00618 | 0.0242 | 0.102 | 0.214 | 6.15 | 99.7% |  |
| PCB 61 + 70 + 74 + 76 | 284 | 0.00116 | 0.0442 | 0.126 | 0.609 | 1.06 | 28.5 | 97.9% |  |
| PCB 63 | 250 | 0.00085 | 0.00182 | 0.00601 | 0.0313 | 0.0706 | 2.23 | 86.2% |  |
| PCB 64 | 282 | 0.00141 | 0.00956 | 0.0337 | 0.165 | 0.512 | 25.9 | 97.2% |  |
| PCB 66 | 285 | 0.00079 | 0.0325 | 0.112 | 0.576 | 1.20 | 70.2 | 98.3% |  |
| PCB 67 | 187 | 0.00086 | < MDL | 0.00166 | 0.00571 | 0.0163 | 0.190 | 64.5% |  |
| PCB 68 | 216 | 0.00080 | < MDL | 0.00182 | 0.00997 | 0.0221 | 0.603 | 74.5% |  |
| PCB 72 | 201 | 0.00081 | < MDL | 0.00159 | 0.0101 | 0.0227 | 0.378 | 69.3% |  |
| PCB 73 | 61 | 0.00136 | < MDL | < MDL | < MDL | 0.00822 | 0.177 | 21.0% |  |
| PCB 77 | 243 | 0.00106 | 0.00141 | 0.00443 | 0.00119 | 0.0255 | 0.384 | 83.8% |  |
| PCB 78 | 7 | 0.00042 | < MDL | < MDL | < MDL | < MDL | 0.00186 | 2.4% |  |
| PCB 79 | 225 | 0.00080 | 0.000839 | 0.00257 | 0.0117 | 0.0265 | 0.677 | 77.6% |  |
| PCB 80 | 1 | 0.00043 | < MDL | < MDL | < MDL | < MDL | 0.000745 | 0.3% |  |
| PCB 81 | 24 | 0.00118 | < MDL | < MDL | < MDL | < MDL | 0.0554 | 8.3% |  |
| PCB 82 | 280 | 0.00086 | 0.00392 | 0.0137 | 0.0465 | 0.116 | 3.18 | 96.6% |  |
| PCB 83 + 99 | 290 | 0.00140 | 0.0784 | 0.224 | 1.12 | 2.66 | 75.7 | 100.0% |  |
| PCB 84 | 277 | 0.00129 | 0.00490 | 0.0142 | 0.0614 | 0.164 | 4.88 | 95.5% |  |
| PCB 85 + 116 + 117 | 290 | 0.00086 | 0.0228 | 0.0712 | 0.301 | 0.724 | 24.4 | 100.0% |  |
| PCB 86 + 87 + 97 + 109 + 119 + 125 | 288 | 0.00140 | 0.0438 | 0.162 | 0.565 | 1.45 | 44.1 | 99.3% |  |
| PCB 88 + 91 | 282 | 0.00129 | 0.00723 | 0.0234 | 0.122 | 0.324 | 13.4 | 97.2% |  |
| PCB 89 | 120 | 0.00076 | < MDL | < MDL | 0.00187 | 0.00557 | 0.277 | 41.4% |  |
| PCB 90 + 101 + 113 | 290 | 0.00140 | 0.0829 | 0.341 | 1.06 | 2.45 | 71.9 | 100.0% |  |
| PCB 92 | 290 | 0.00103 | 0.0174 | 0.0550 | 0.262 | 0.589 | 14.5 | 100.0% |  |
| PCB 93 + 95 + 98 + 100 + 102 | 286 | 0.00129 | 0.0281 | 0.104 | 0.374 | 0.874 | 24.6 | 98.6% |  |
| PCB 94 | 121 | 0.00094 | < MDL | < MDL | 0.00267 | 0.00805 | 0.363 | 41.7% |  |
| PCB 96 | 79 | 0.00124 | < MDL | < MDL | < MDL | 0.00387 | 0.128 | 27.2% |  |
| PCB 103 | 179 | 0.00116 | < MDL | 0.00229 | 0.00949 | 0.0303 | 1.27 | 61.7% |  |
| PCB 104 | 42 | 0.00084 | < MDL | < MDL | < MDL | 0.00109 | 0.0958 | 14.5% |  |
| PCB 105 | 290 | 0.00132 | 0.0408 | 0.149 | 0.499 | 1.15 | 18.6 | 100.0% |  |
| PCB 106 | 1 | 0.00073 | < MDL | < MDL | < MDL | < MDL | 0.00176 | 0.3% |  |
| PCB 107 | 290 | 0.00048 | 0.0101 | 0.0351 | 0.115 | 0.261 | 4.65 | 100.0% |  |
| PCB 108 + 124 | 283 | 0.00048 | 0.00321 | 0.0116 | 0.0311 | 0.0563 | 1.22 | 97.6% |  |
| PCB 110 + 115 | 287 | 0.00086 | 0.0760 | 0.276 | 1.08 | 2.33 | 78.1 | 99.0% |  |
| PCB 111 | 153 | 0.00058 | < MDL | < MDL | 0.00263 | 0.00567 | 0.214 | 52.8% |  |
| PCB 112 | 0 | 0.00140 | < MDL | < MDL | < MDL | < MDL | 0.00 | 0.0% |  |
| PCB 114 | 258 | 0.00130 | 0.00269 | 0.00913 | 0.0326 | 0.0837 | 1.05 | 89.0% |  |
| PCB 118 | 290 | 0.00118 | 0.118 | 0.462 | 1.37 | 3.64 | 56.8 | 100.0% |  |
| PCB 120 | 240 | 0.00070 | 0.00101 | 0.00262 | 0.0130 | 0.0303 | 0.659 | 82.8% |  |
| PCB 121 | 108 | 0.00067 | < MDL | < MDL | 0.00154 | 0.00366 | 0.113 | 37.2% |  |
| PCB 122 | 242 | 0.00023 | 0.000742 | 0.00274 | 0.00975 | 0.0237 | 0.603 | 83.4% |  |
| PCB 123 | 208 | 0.00126 | < MDL | 0.00493 | 0.0224 | 0.0711 | 1.45 | 71.7% |  |
| PCB 126 | 75 | 0.00133 | < MDL | < MDL | < MDL | 0.00593 | 0.0856 | 25.9% |  |
| PCB 127 | 179 | 0.00024 | < MDL | 0.000789 | 0.00435 | 0.0106 | 0.165 | 61.7% |  |
| PCB 128 + 166 | 290 | 0.00085 | 0.0338 | 0.126 | 0.475 | 0.983 | 12.9 | 100.0% |  |
| PCB 129 + 138 + 160 + 163 | 290 | 0.00085 | 0.277 | 0.896 | 3.94 | 9.11 | 156 | 100.0% |  |
| PCB 130 | 289 | 0.00113 | 0.0119 | 0.0427 | 0.166 | 0.381 | 5.73 | 99.7% |  |
| PCB 131 | 214 | 0.00076 | < MDL | 0.00264 | 0.0107 | 0.0264 | 0.458 | 73.8% |  |
| PCB 132 | 289 | 0.00113 | 0.0176 | 0.0731 | 0.274 | 0.706 | 15.2 | 99.7% |  |
| PCB 133 | 285 | 0.00075 | 0.00485 | 0.0131 | 0.0651 | 0.149 | 3.41 | 98.3% |  |
| PCB 134 + 143 | 267 | 0.00102 | 0.000283 | 0.0103 | 0.0370 | 0.109 | 2.01 | 92.1% |  |
| PCB 135 + 151 + 154 | 289 | 0.00102 | 0.0342 | 0.120 | 0.532 | 1.45 | 30.0 | 99.7% |  |
| PCB 136 | 263 | 0.00130 | 0.00340 | 0.0137 | 0.0476 | 0.115 | 3.27 | 90.7% |  |
| PCB 137 | 290 | 0.00080 | 0.0109 | 0.0370 | 0.126 | 0.309 | 4.04 | 100.0% |  |
| PCB 139 + 140 | 277 | 0.00076 | 0.00305 | 0.00895 | 0.0451 | 0.0905 | 1.27 | 95.5% |  |
| PCB 141 | 290 | 0.00152 | 0.0233 | 0.0876 | 0.336 | 1.03 | 18.8 | 100.0% |  |
| PCB 142 | 0 | 0.00117 | < MDL | < MDL | < MDL | < MDL | 0.00 | 0.0% |  |
| PCB 144 | 276 | 0.00102 | 0.00352 | 0.0139 | 0.0541 | 0.160 | 2.95 | 95.2% |  |
| PCB 145 | 4 | 0.00169 | < MDL | < MDL | < MDL | < MDL | 0.00649 | 1.4% |  |
| PCB 146 | 290 | 0.00100 | 0.0537 | 0.152 | 0.727 | 1.61 | 27.7 | 100.0% |  |
| PCB 147 + 149 | 290 | 0.00102 | 0.0846 | 0.290 | 1.39 | 3.37 | 73.5 | 100.0% |  |
| PCB 148 | 165 | 0.00084 | < MDL | 0.00106 | 0.00679 | 0.0168 | 0.372 | 56.9% |  |
| PCB 150 | 107 | 0.00139 | < MDL | < MDL | 0.00228 | 0.00672 | 0.271 | 36.9% |  |
| PCB 152 | 40 | 0.00135 | < MDL | < MDL | < MDL | 0.00165 | 0.0779 | 13.8% |  |
| PCB 153 + 168 | 290 | 0.00152 | 0.313 | 0.908 | 4.24 | 9.92 | 172 | 100.0% |  |
| PCB 155 | 138 | 0.00100 | < MDL | < MDL | 0.00332 | 0.0118 | 0.899 | 47.6% |  |
| PCB 156 + 157 | 290 | 0.00136 | 0.0223 | 0.0800 | 0.260 | 0.733 | 7.88 | 100.0% |  |
| PCB 158 | 290 | 0.00085 | 0.0200 | 0.0688 | 0.276 | 0.679 | 9.10 | 100.0% |  |
| PCB 159 | 211 | 0.00084 | < MDL | 0.00343 | 0.0173 | 0.0470 | 1.72 | 72.8% |  |
| PCB 161 | 0 | 0.00115 | < MDL | < MDL | < MDL | < MDL | 0.00 | 0.0% |  |
| PCB 162 | 244 | 0.00076 | 0.00110 | 0.00313 | 0.0114 | 0.0281 | 0.492 | 84.1% |  |
| PCB 164 | 290 | 0.00096 | 0.0111 | 0.0376 | 0.154 | 0.405 | 6.14 | 100.0% |  |
| PCB 165 | 90 | 0.00088 | < MDL | < MDL | 0.00147 | 0.00536 | 0.316 | 31.0% |  |
| PCB 167 | 287 | 0.00136 | 0.0123 | 0.0411 | 0.142 | 0.394 | 4.51 | 99.0% |  |
| PCB 169 | 5 | 0.00102 | < MDL | < MDL | < MDL | < MDL | 0.00554 | 1.7% |  |
| PCB 170 | 290 | 0.00077 | 0.0544 | 0.150 | 0.645 | 1.90 | 33.3 | 100.0% |  |
| PCB 171 + 173 | 286 | 0.00121 | 0.0153 | 0.0400 | 0.224 | 0.698 | 8.67 | 98.6% |  |
| PCB 172 | 289 | 0.00091 | 0.0136 | 0.0379 | 0.144 | 0.533 | 8.75 | 99.7% |  |
| PCB 174 | 290 | 0.00125 | 0.0255 | 0.0892 | 0.399 | 1.51 | 30.2 | 100.0% |  |
| PCB 175 | 248 | 0.00130 | 0.00213 | 0.00649 | 0.0328 | 0.102 | 1.19 | 85.5% |  |
| PCB 176 | 245 | 0.00135 | 0.00204 | 0.00714 | 0.0386 | 0.0842 | 3.19 | 84.5% |  |
| PCB 177 | 290 | 0.00084 | 0.0183 | 0.0698 | 0.289 | 1.00 | 27.2 | 100.0% |  |
| PCB 178 | 290 | 0.00074 | 0.0150 | 0.0427 | 0.187 | 0.537 | 12.3 | 100.0% |  |
| PCB 179 | 283 | 0.00108 | 0.00746 | 0.0246 | 0.123 | 0.277 | 8.66 | 97.6% |  |
| PCB 180 + 193 | 290 | 0.00079 | 0.182 | 0.506 | 2.25 | 6.67 | 112 | 100.0% |  |
| PCB 181 | 179 | 0.00121 | < MDL | 0.00190 | 0.00808 | 0.0175 | 0.234 | 61.7% |  |
| PCB 182 | 151 | 0.00138 | < MDL | < MDL | 0.00541 | 0.0129 | 0.312 | 52.1% |  |
| PCB 183 + 185 | 290 | 0.00138 | 0.0411 | 0.113 | 0.629 | 2.11 | 30.5 | 100.0% |  |
| PCB 184 | 141 | 0.00110 | < MDL | < MDL | 0.00340 | 0.00988 | 0.837 | 48.6% |  |
| PCB 186 | 1 | 0.00124 | < MDL | < MDL | < MDL | < MDL | 0.00151 | 0.3% |  |
| PCB 187 | 290 | 0.00111 | 0.126 | 0.377 | 1.89 | 5.62 | 80.0 | 100.0% |  |
| PCB 188 | 131 | 0.00102 | < MDL | < MDL | 0.00352 | 0.00650 | 0.170 | 45.2% |  |
| PCB 189 | 280 | 0.00060 | 0.00271 | 0.00727 | 0.0248 | 0.0822 | 1.70 | 96.6% |  |
| PCB 190 | 289 | 0.00075 | 0.0150 | 0.0411 | 0.183 | 0.617 | 8.81 | 99.7% |  |
| PCB 191 | 267 | 0.00083 | 0.00250 | 0.00723 | 0.0309 | 0.101 | 1.53 | 92.1% |  |
| PCB 192 | 5 | 0.00079 | < MDL | < MDL | < MDL | < MDL | 0.0135 | 1.7% |  |
| PCB 194 | 290 | 0.00083 | 0.0336 | 0.0936 | 0.387 | 1.28 | 27.0 | 100.0% |  |
| PCB 195 | 287 | 0.00101 | 0.0121 | 0.0299 | 0.147 | 0.441 | 7.05 | 99.0% |  |
| PCB 196 | 288 | 0.00121 | 0.0141 | 0.0385 | 0.181 | 0.566 | 8.23 | 99.3% |  |
| PCB 197 + 200 | 275 | 0.00093 | 0.00284 | 0.00832 | 0.0408 | 0.0973 | 2.47 | 94.8% |  |
| PCB 198 + 199 | 290 | 0.00121 | 0.0467 | 0.134 | 0.505 | 1.69 | 25.4 | 100.0% |  |
| PCB 201 | 275 | 0.00121 | 0.00346 | 0.0102 | 0.0507 | 0.147 | 2.97 | 94.8% |  |
| PCB 202 | 288 | 0.00114 | 0.0110 | 0.0333 | 0.120 | 0.390 | 4.89 | 99.3% |  |
| PCB 203 | 290 | 0.00104 | 0.0322 | 0.0853 | 0.376 | 1.18 | 18.6 | 100.0% |  |
| PCB 204 | 51 | 0.00093 | < MDL | < MDL | < MDL | 0.00154 | 0.0731 | 17.6% |  |
| PCB 205 | 249 | 0.00103 | 0.00191 | 0.00500 | 0.0177 | 0.0676 | 0.841 | 85.9% |  |
| PCB 206 | 290 | 0.00111 | 0.0279 | 0.0688 | 0.241 | 0.646 | 18.3 | 100.0% |  |
| PCB 207 | 231 | 0.00269 | 0.00320 | 0.00850 | 0.0294 | 0.0791 | 1.80 | 79.7% |  |
| PCB 208 | 289 | 0.00099 | 0.00958 | 0.0267 | 0.0987 | 0.205 | 8.14 | 99.7% |  |
| PCB 209 | 290 | 0.00096 | 0.0165 | 0.0436 | 0.152 | 0.375 | 21.2 | 100.0% |  |
| **Total PCBs** | **290** |  | **2.52** | **9.04** | **35.3** | **94.8** | **1212** | **100.0%** |  |
| ^a^ MDL= Method Detection Limit in ng/g, wet weight  ^b^ Percentile concentrations are statistical estimates for the sampled population of river length determined for the study (a length of 66,142 km); maximum concentrations are the maximum values measured at 290 sites.  ^c^ Percent frequency of occurrence values are based on 290 possible detections. | | | | | | | | | |

| **Table A.13. PFAS results (ng/g, wet weight) for 2013–14 NRSA fish fillet tissue samples.** | | | | | | | | |
| --- | --- | --- | --- | --- | --- | --- | --- | --- |
| **Chemical** | **Number of detections** | **MDL^a^** | **Percentile^b^** | | | | **Maximum** | **Frequency of occurrence^c^** |
|  |  |  | **25^th^** | **50^th^** | **75^th^** | **90^th^** |  |  |
| PFBA | 29 | 0.100 | < MDL | < MDL | < MDL | < MDL | 48.1 | 8.3% |
| PFBS | 3 | 0.100 | < MDL | < MDL | < MDL | < MDL | 0.571 | 0.9% |
| PFPeA | 11 | 0.069 | < MDL | < MDL | < MDL | < MDL | 0.884 | 3.2% |
| PFHxA | 32 | 0.052 | < MDL | < MDL | < MDL | < MDL | 1.44 | 9.2% |
| PFHxS | 32 | 0.066 | < MDL | < MDL | < MDL | 0.0252 | 0.980 | 9.2% |
| PFHpA | 1 | 0.060 | < MDL | < MDL | < MDL | < MDL | 0.660 | 0.3% |
| PFOA | 15 | 0.110 | < MDL | < MDL | < MDL | < MDL | 0.271 | 4.3% |
| PFOS | 346 | 0.077 | 2.85 | 6.49 | 18.8 | 43.9 | 283 | 99.1% |
| PFOSA | 79 | 0.071 | < MDL | < MDL | < MDL | 0.369 | 35.0 | 22.6% |
| PFNA | 135 | 0.043 | < MDL | < MDL | 0.267 | 0.660 | 1.91 | 38.7% |
| PFDA | 293 | 0.073 | 0.226 | 0.580 | 1.16 | 2.12 | 18.0 | 84.0% |
| PFUnA | 308 | 0.074 | 0.317 | 0.621 | 1.37 | 2.56 | 53.9 | 88.3% |
| PFDoA | 244 | 0.059 | < MDL | 0.308 | 0.742 | 1.90 | 99.5 | 69.9% |
| ^a^ MDL= Method Detection Limit in ng/g, wet weight  ^b^ Percentile concentrations are statistical estimates for the sampled population of river length determined for the study (a length of 78,272 km); maximum concentrations are the maximum values measured at 349 sites.  ^c^ Percent frequency of occurrence values are based on 349 possible detections. | | | | | | | | |

| **Table A.14. PFAS results (ng/g, wet weight) for 2018–19 NRSA fish fillet tissue samples.** | | | | | | | | |
| --- | --- | --- | --- | --- | --- | --- | --- | --- |
| **Chemical** | **Number of detections** | **MDL^a^** | **Percentile^b^** | | | | **Maximum** | **Frequency of occurrence^c^** |
|  |  |  | **25^th^** | **50^th^** | **75^th^** | **90^th^** |  |  |
| PFBA | 18 | 0.551 | < MDL | < MDL | < MDL | < MDL | 0.806 | 6.2% |
| PFPeA | 0 | 0.192 | < MDL | < MDL | < MDL | < MDL | 0.00 | 0.0% |
| PFHxA | 0 | 0.203 | < MDL | < MDL | < MDL | < MDL | 0.00 | 0.0% |
| PFHpA | 0 | 0.170 | < MDL | < MDL | < MDL | < MDL | 0.00 | 0.0% |
| PFOA | 6 | 0.162 | < MDL | < MDL | < MDL | < MDL | 0.354 | 2.1% |
| PFNA | 120 | 0.129 | < MDL | < MDL | 0.176 | 0.273 | 1.44 | 41.4% |
| PFDA | 256 | 0.116 | 0.200 | 0.332 | 0.655 | 1.23 | 29.8 | 88.3% |
| PFUnA | 246 | 0.151 | 0.237 | 0.516 | 0.939 | 1.52 | 105 | 84.8% |
| PFDoA | 201 | 0.156 | < MDL | 0.277 | 0.533 | 1.30 | 140 | 69.3% |
| PFTrDA | 161 | 0.398 | < MDL | 0.421 | 0.845 | 1.52 | 140 | 55.5% |
| PFTeDA | 105 | 0.309 | < MDL | < MDL | 0.390 | 0.854 | 62.8 | 36.2% |
| PFBS | 0 | 0.097 | < MDL | < MDL | < MDL | < MDL | 0.00 | 0.0% |
| PFPeS | 0 | 0.129 | < MDL | < MDL | < MDL | < MDL | 0.00 | 0.0% |
| PFHxS | 6 | 0.153 | < MDL | < MDL | < MDL | < MDL | 0.611 | 2.1% |
| PFHpS | 1 | 0.154 | < MDL | < MDL | < MDL | < MDL | 0.162 | 0.34% |
| PFOS | 265 | 0.354 | 1.15 | 3.07 | 7.97 | 18.2 | 131 | 91.4% |
| PFNS | 1 | 0.155 | < MDL | < MDL | < MDL | < MDL | 0.224 | 0.34% |
| PFDS | 88 | 0.207 | < MDL | < MDL | 0.225 | 0.497 | 4.97 | 30.3% |
| PFDoS | 0 | 0.291 | < MDL | < MDL | < MDL | < MDL | 0.00 | 0.0% |
| 4:2FTS | 0 | 0.234 | < MDL | < MDL | < MDL | < MDL | 0.00 | 0.0% |
| 6:2FTS | 14 | 0.404 | < MDL | < MDL | < MDL | < MDL | 12.1 | 4.8% |
| 8:2FTS | 0 | 0.670 | < MDL | < MDL | < MDL | < MDL | 0.00 | 0.0% |
| PFOSA | 69 | 0.152 | < MDL | < MDL | < MDL | 0.250 | 2.87 | 23.8% |
| N-MeFOSA | 0 | 0.288 | < MDL | < MDL | < MDL | < MDL | 0.00 | 0.0% |
| N-EtFOSA | 0 | 0.248 | < MDL | < MDL | < MDL | < MDL | 0.00 | 0.0% |
| N-MeFOSAA | 7 | 0.304 | < MDL | < MDL | < MDL | < MDL | 0.756 | 2.4% |
| N-EtFOSAA | 10 | 0.143 | < MDL | < MDL | < MDL | < MDL | 1.38 | 3.4% |
| N-MeFOSE | 0 | 3.360 | < MDL | < MDL | < MDL | < MDL | 0.00 | 0.0% |
| N-EtFOSE | 16 | 1.450 | < MDL | < MDL | < MDL | < MDL | 3.62 | 5.5% |
| HFPO-DA | 0 | 0.460 | < MDL | < MDL | < MDL | < MDL | 0.00 | 0.0% |
| ADONA | 0 | 0.884 | < MDL | < MDL | < MDL | < MDL | 0.00 | 0.0% |
| 9Cl-PF3ONS | 0 | 0.708 | < MDL | < MDL | < MDL | < MDL | 0.00 | 0.0% |
| 11Cl-PF3OUdS | 0 | 0.889 | < MDL | < MDL | < MDL | < MDL | 0.00 | 0.0% |
| ^a^ MDL= Method Detection Limit in ng/g, wet weight  ^b^ Percentile concentrations are statistical estimates for the sampled population of river length determined for the study (a length of 66,142 km); maximum concentrations are the maximum values measured at 290 sites.  ^c^ Percent frequency of occurrence values are based on 290 possible detections. | | | | | | | | |
